# A methodological approach to identify agro-biodiversity hotspots for priority *in situ* conservation of plant genetic resources

**DOI:** 10.1371/journal.pone.0197709

**Published:** 2018-06-01

**Authors:** Luca Pacicco, Mara Bodesmo, Renzo Torricelli, Valeria Negri

**Affiliations:** Dipartimento di Scienze Agrarie, Alimentari e Ambientali (DSA3), Università degli Studi di Perugia, Perugia, Italy; Brigham Young University, UNITED STATES

## Abstract

Agro-biodiversity is seriously threatened worldwide and strategies to preserve it are dramatically required. We propose here a methodological approach aimed to identify areas with a high level of agro-biodiversity in which to set or enhance *in situ* conservation of plant genetic resources. These areas are identified using three criteria: Presence of Landrace diversity, Presence of wild species and Agro-ecosystem ecological diversity. A Restrictive and an Additive prioritization strategy has been applied on the entire Italian territory and has resulted in establishing nationwide 53 and 197 agro-biodiversity hotspots respectively. At present the strategies can easily be applied at a European level and can be helpful to develop conservation strategies everywhere.

## Introduction

‘Biological diversity’ exists at three main levels: the diversity within species, between species and of ecosystems [1]. Over the last decade the scientific community has been stressing the importance of biodiversity conservation, which has been prompted by increasing biodiversity loss worldwide [2–4]. In 2002, the Parties to the Convention on Biological Diversity [5] set the goal to reduce the rate of biodiversity loss by the year 2010, but it has been widely acknowledged that this target has not been met [6].

On the basis of number of endemic plants and threat to original natural vegetation, the World Wildlife Fund and Conservation International [7] has identified 35 regions as biodiversity “hotspots”, i.e. areas biologically rich and seriously threatened which must be protected. Among them the Mediterranean Basin is one of the most important biodiversity hotspots [8]. In fact it includes about 25,000 plant species (of which around 13,000 are endemic), 4.3% of global plant species, estimated at 300,000 [4], endemic amphibians, reptiles, birds and mammals. Referring to plants in particular, the Mediterranean area is a hotspot not only for wild species diversity, but also for cultivated plant diversity [9].

The concern for biodiversity loss should also include the concern for agro-biodiversity loss, i.e. concern for the loss of agro-ecosystems, loss of species and of populations within species that are important for agriculture. Agro-ecosystems are human managed ecosystems that include all components of biological diversity of relevance to food and agriculture [10] and are dominant forms of ecosystems globally, covering nearly 40 per cent of the terrestrial surface of the Earth, and especially in densely inhabited regions, such as Europe [11]. Agro-ecosystems provide humans with food, forage, fibre, fuel, furniture, pharmaceuticals and cultural services (such as scenic beauty, education, recreation and tourism, traditional use, rituals and customs that bond human communities) that are essential to their wellbeing.

There is a continuous trade-off between agro-ecosystems and ‘natural’ ecosystems [12]. Agro-ecosystems provide for and depend on many ‘natural’ ecosystem services. For example, crop production depends on supporting services such as pollinators, nutrients, biological pest control, maintenance of soil structure and fertility, cycling and hydrological services [12], but it also influences regulating services that provide benefits external to farms such as soil and water quality regulation, carbon sequestration, and protection and enhancement of biodiversity [12–15]. These also quantify and maintain ecosystem services, for example, through high species diversity [16], and contribute towards multifunctional agricultural systems [17].

Scientists refer to that part of between- and within-species plant diversity that is used by mankind in agriculture as “plant genetic resources”. Beside others (commercial and obsolete varieties, genetic stocks, breeding lines) they include the wild progenitors and the landraces (also called “farmer varieties,” “local varieties” or “primitive varieties”) of the crops. Crop wild relatives are wild plant species that are genetically related to cultivated crops [18,19] while landraces are variable, identifiable populations, lacking ‘formal’ crop improvement, characterized by a specific adaptation to the environmental conditions of the area of cultivation and by a close association with traditional uses, knowledge, habits, dialects, and celebrations of the people who developed and continue to grow them [20]. Crop wild relative and landrace populations are a critical source of genes that allow crops to adapt to ever-changing conditions and to overcome the constraints caused by pests, diseases and abiotic stresses; they consequently are essential for sustainable agricultural production and food security in a scenario of climate change and unpredictability. Crop wild relatives and landraces are the most threatened among plant genetic resources and deserve to be conserved with priority [21,22].

It is to be noted that twenty two years after the landmark 1992 Earth Summit in Rio and the CBD, 1992 [1], the Rio+20 declaration on ‘The future we want’ reaffirmed the need to improve food security based on sustainable agricultural practices that preserve natural resources, which of course includes genetic resources [23]. Genetic erosion and sustainable use of crop genetic resources, and the fair and equitable sharing of benefits deriving from them, are in the focus of the targets adopted by the FAO Commission on Genetic Resources for Food and Agriculture [24], the Second Global Plan of Action for Plant Genetic Resources for Food and Agriculture [11], The International Treaty on Plant Genetic Resources for Food and Agriculture [25] and the Nagoya Protocol [26].

A specific agro-biodiversity conservation plan is needed for Europe because the region includes the Mediterranean basin biodiversity hotspot and is rich in both crop wild relatives and landraces that are still cultivated [22, 27–36], while high population density, widespread industrial and agricultural activities and the effects of climate change make biodiversity vulnerable. In fact, as can be seen from data of the European Union which covers a large part of Europe, and in spite of the European Union network of protected areas, the existence of quite detailed information systems on nature conservation, and of common agro-environmental policies, biodiversity decline has not been halted in recent years (European Parliament Resolution 2016) [37].

In this scenario, it is important to adopt a “systematic conservation planning” process to identify areas with a high level of biodiversity [38,39], taking into account local, ecological, social, economic, political and cultural factors and identifying areas in which to program conservation action over extensive time periods [39]. The formulation of effective and efficient conservation strategies requires a thorough understanding of spatial patterns of genetic diversity [40] where geographical information system (GIS) could play a fundamental role in facilitating more detailed and accurate analyses.

Several studies have been carried out on the subject which have taken into account the genetic diversity of a certain species [41], species number evaluated through a series of indices [42,43], ecological diversity [44] or other elements such as distributions of species [45] or economic value of biodiversity [46]. In addition, a multi-criteria approach, which includes ecological criteria (i.e. richness, rarity, endemism), socioeconomic criteria (i.e. ecosystem services, recreation actions, future economic value) and management history criteria (management, monitoring, specific environmental studies, environmental awareness, education, research) has also been used [44].

Concerning the tools used, Paracchini et al. [47] identified High Nature Value Areas using Land cover data, the CORINE data base, CLC 2006 [48]. Other authors have identified the best sites for plant conservation using tools like GIS and ecogeographic maps of a selection of vascular plants, bryophytes, fresh water algae, lichens or fungi [49], and in some cases have also applied a gap analysis, a useful method to develop strategies when conservation of genetic diversity is the prime goal [50–54].

However none of these studies has ever considered the identification of agro-biodiversity hotspots by globally taking into account all their different components.

The aim of this paper was to set a methodological approach to systematic conservation planning in Europe which can identify agro-biodiversity hotspots, hereafter referred to as Most Appropriate Areas (MAPAs), in which to set or enhance already present *in situ* conservation activities for plant genetic resources.

To this purpose, the main characteristics of the systematic conservation planning process (i.e. explicit goals, clear choices and simple processes [38]) were considered: i) as an explicit goal, we considered all the components of agro-biodiversity (agro-ecosystem ecological diversity, specific and intraspecific diversity of landraces and presence of wild species) ii) we operated clear choices about the features to be used as proxies for overall biodiversity on the basis of previous discussions among experts and tests [55] and iii) we used simple processes to prioritize areas and identify MAPAs.

## Material and methods

### 1. The sample area used for the methodological approach

The methodology was developed by taking as an example a specific area of the Mediterranean hotspot for which sufficient data on agro-ecosystem ecological diversity, landraces and presence of wild species exist (i.e. Italy).

Italy is one of the richest countries in the European Union for biodiversity having i) the highest density of wild plants and animals of the entire Europe [56–60], ii) 2,549 Sites of Community Importance (SCIs) and Special Protection Areas (SPAs), the latter covering 21.6% of the national territory (62,623 km^2^) [61], and iii) hundreds of landraces [32].

In this study the Italian area was subdivided into 995 quadrants by merging the International Union for Conservation of Nature [62] 2x2 km grids into 20x20 km grids (i.e. 400 km^2^ quadrants), each quadrant being georeferenced using the WGS84-UTM32N system (EPSG: 32632) (“S1 Table”).

### 2. Criteria used to assess agro-biodiversity components

In order to define and identify diversity-rich areas, three criteria were chosen following those already discussed by experts and tested in a previous study [55].

### 2.1. Criterion 1 (C1)–landrace diversity (LRD)

As mentioned above, landraces are an important component of agro-biodiversity to be protected with priority [63,64]. Contrary to most commercial cultivars (generally pure lines or hybrids) that are composed of a sole genotype, landraces are characterized by intrinsic diversity (i.e. are composed of different genotypes [65–69], just to mention some of many references). As Esquinas-Alcazar writes [70] “The heterogeneous varieties of the past have been and still are the plant breeder’s raw material. They have been a fruitful, sometimes the sole, source of genes for pest and disease resistance, adaptation to difficult environments, and other agricultural traits like the dwarf-type in cereals that have contributed to the green revolution in many parts of the world”. This is particularly important in a period of climate change and unpredictability. Data relative to this criterion were extracted from a landrace inventory for Italy [32]. This inventory includes 4,803 georeferenced accessions of 2,365 landraces belonging to 329 different species, and was compiled from official information retrieved from each Italian Region (the Public bodies which are responsible for plant genetic resources conservation in Italy).

### 2.2. Criterion 2 (C2)—presence of wild species (PWS)

It is obviously not feasible to have data on all the species present in a certain area. As a consequence, and following previous experiences [55], we proxy estimated this component of agro-biodiversity through the presence of protected areas. This is because protected areas i) are generally set where a high number of species or rare/endemic species exist and ii) provide supporting services (nutrient recycling, primary production and soil formation), provision services (production of food and shelter for pollinators and other useful organisms and of water for all living beings), and regulating services (control of climate, of air and water quality, of pests and diseases) which are of value for agro-ecosystems.[71]

In addition, the presence of a certain protected area can positively influence the future setting of other areas by enhancing the already-existing circuits of natural tourism/initiatives related to the protection of nature or the implementation of sustainable agriculture [55].

Finally, some protected areas also contain wild relatives of important crops [36,72]. In particular in Italy, the wild relatives of the crops listed in Annex 1 of the International Treaty on Plant Genetic Resources for Food and Agriculture [25] were recently recorded for National Parks, Natura 2000 sites and other protected areas (see the Network Nazionale Biodiversità web site [72]).

### 2.3. Criterion 3 (C3)—Agro-ecosystem ecological diversity (AED)

Agro-ecosystem ecological diversity is an essential criterion to be considered since i) it is associated with the number of species and the level of inter and intra-population diversity of each species and ii) determines the complexity level of relationships among living beings [73,74]. On the basis of previous discussions and data working out [55], we chose a proxy estimate for this criterion as well: the percentage of territory covered by agricultural areas, forests, seminatural areas, wetlands and water bodies. The idea is that any territory different from artificial areas contains a certain level of biodiversity. The higher the number of different uses of a certain territory, the higher its biodiversity should be. Data relative to territory use in a certain quadrant were worked out using the Corine Land Cover land cover/land use map [48], a tool that is available online for the entire European territory, which then makes it possible to extend the use of this criterion regionally.

### 3. Index calculation per unit area

In order to apply the criteria, for each unit area (i.e. each single 400 km^2^ quadrant) the following indices were calculated.

- In order to apply criterion 1 (LRD) the Italian Landrace Inventory [32], where for each landrace latitude and longitude are reported, was used. Initially, a landrace number per quadrant, i.e. a Landrace Density Index (LDI) was calculated. Then, for those quadrants with LDI≥1, an estimate of diversity per quadrant, i.e. a Shannon Diversity Index (H’) [75,76] was calculated as:

$$H^{\prime }=-\overset{s}{\underset{i=1}{\sum }}p_{i}*\text{ln}p_{i}$$

where *i* is the number of species cultivated as landraces and *p*_*i*_ is the landrace frequency, i.e. number of landraces belonging to each species/landrace total number.

To apply criterion 2 (PWS) the cartographic information that is available from the Elenco Ufficiale delle Aree Naturali Protette (EUAP [77]) and Natura 2000 Network [61] was used. EUAP is the Official National Inventory of Protected Areas that includes all natural areas established in accordance with national law (i.e. National and Regional Parks and national reserves) [78]. The Natura 2000 Network [61] stretches over 18% of the EU’s land area and almost 6% of its marine territory, making it the largest coordinated network of protected areas in the world. It offers a haven to Europe's most valuable and threatened species and habitats [61]. It provides information on protected sites that support rare, endangered or vulnerable natural habitats and species of plants or animals, areas designated under the Habitats Directive [79] and areas supporting significant numbers of wild birds and their habitats (protected sites designated under the Birds Directive [80]). For both, information is provided as visual layers. Overlapping the EUAP and the Natura 2000 Network layers, a unique ‘EUAP_N2N layer’ was obtained containing all the needed information. For each quadrant the percentage area covered by protected areas was calculated.

Finally, to apply criterion 3 (AED) the most recent version of the Corine Land Cover-CLC [48], which is based on satellite photographs, was used.

CLC 2006 contains 5 main categories (named Level 1) of land cover types: 1 artificial areas, 2 agricultural land, 3 forests and semi-natural areas, 4 wetlands, 5 water surfaces), each category is subdivided into a different number of sub-categories (Level 2) and these latter sub-categories are subdivided into sub-sub-categories (Level 3) (“S2 Table”). In this study the land cover categories Level 2, 3, 4 and 5 with all their subdivisions (Levels 2 and 3) were considered. For each quadrant the percentage area covered by all of them was worked out.

### 4. Two prioritization strategies applied

For each one of the 995 quadrants information on LRD, PWS and AED was obtained applying the above-mentioned indices (“S1 Table”). The next step was to prioritize these areas and identify MAPAs. To that purpose two prioritization strategies were defined: a Restrictive Strategy (RS) and an Additive Strategy (AS).

**Fig 1:**
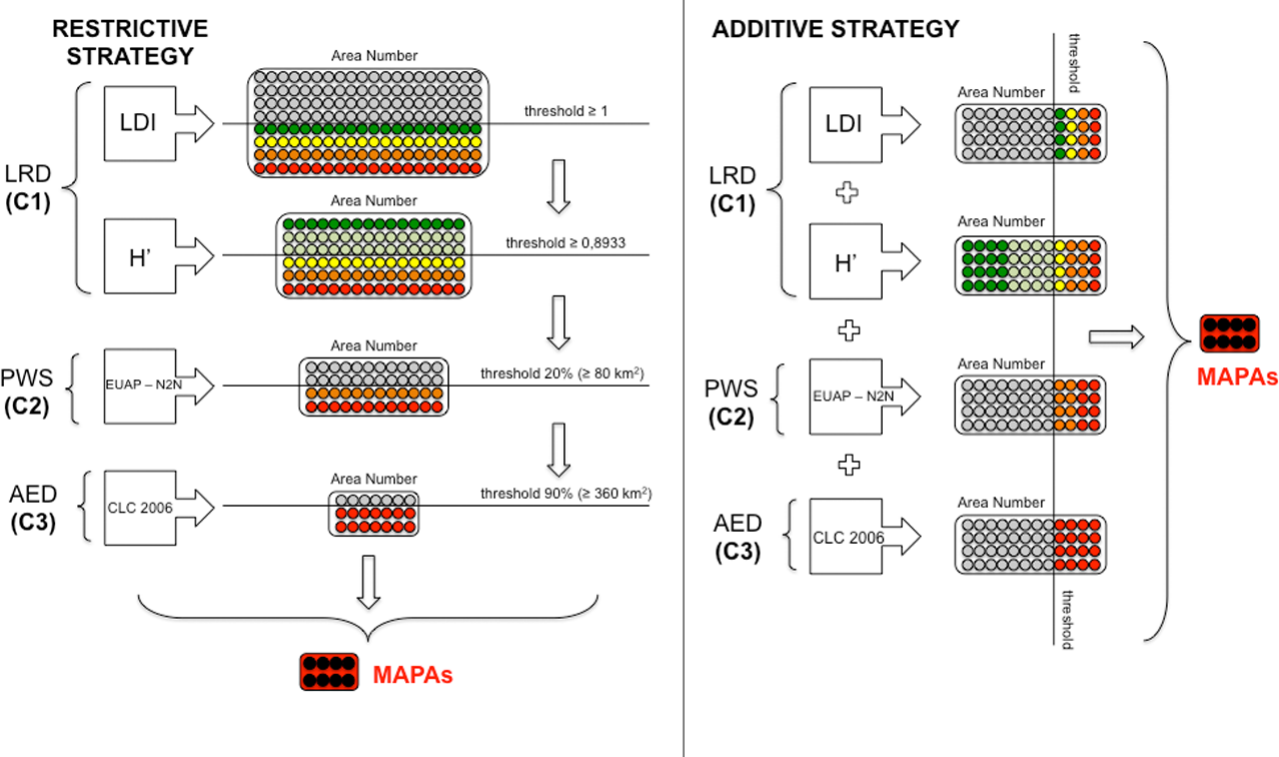
The two prioritization strategies used to identify MAPAs for agro-biodiversity conservation. Left: the Restrictive Strategy where for each step a threshold was defined, below which areas were not admitted to the following level. Only the areas that passed all the sieving stages were selected as MAPAs. Right: the Additive Strategy where a general threshold was initially worked out and only areas with value equal to or above it were selected as MAPAs. See the text for further explanations.

In the RS (Fig 1 left) the above-mentioned criteria were applied step by step after having decided a threshold value (see Table 1) for each of the indices used to estimate agro-biodiversity level for unit area; below the threshold a certain area was not admitted to the following step (area discrimination, Table 1). In other words, the passage from one criterion to the next took place after having excluded the quadrants with a value lower than that of the threshold: only the areas that passed all the sieving stages of the process were finally identified as MAPAs. It should be noted that whatever the criterion order was, the final result was the same.

**Table 1:** Threshold values for each index used to apply the RS and the AS prioritization strategies.

Criterion	Index	Thresholds	
		Restrictive Strategy	Additive Strategy (normalised data)
C1 –LRD^a^	*LDI*^b^	≥ 1	3.2
	*H'*^c^	≥ 0.893	36.5
C2 –PWS^d^	*EUAP-N2N*^e^	≥ 20% (≥ 80 km^2^)	71.6
C3 –AED^f^	*CLC–L 2+3+4+5* ^g^	≥ 90% (≥ 360 km^2^)	26.6
			**Total 137.9**

^a^ Criterion 1—Landrace diversity

^b^ Landrace Density Index

^c^ Shannon Index

^d^ Criterion 2—Presence of wild species under threat

e Elenco Ufficiale delle Aree Naturali Protette (Ministero dell'Ambiente, della Tutela del Territorio e del Mare) and Natura 2000 Network (European Commission)

^f^ Agro-ecosystem Ecological Diversity

^g^ Corine Land Cover, level 2,3,4,5

The sum of their normalised values was used in the AS prioritization strategy.

In the AS (Fig 1 right) the same threshold values (see Table 1) for each of the indices used to estimate agro-biodiversity level for unit area were used, but in a different manner since values were summed up to obtain a general threshold. To that purpose each threshold value was initially standardized through an arcsine transformation [81]. This made it possible to stabilize variances and normalize proportional data so as to be able to sum them (see below). Only those quadrants with total index values equal to or above the threshold were selected as MAPAs.

### 5. Thresholds applied in the RS and AS prioritization strategies

In the RS prioritization strategy, in order to admit/not admit a certain area to the following level, for each index used to estimate biodiversity components, thresholds were “arbitrarily” defined. Nevertheless, in the definition of the thresholds, we considered the need not to greatly reduce the number of quadrants *ab initio* (and then allow the identification of MAPAs across all the Italian Regions, considering that the Regions are the responsible authorities for agro-biodiversity conservation in Italy) and the fact that the Italian territory shows a wide geographic and ecological heterogeneity (in other words the need to adapt the strategy to this so diverse territory). As such non-stringent thresholds were defined as follows.

Concerning Criterion 1 (LRD) only the quadrants having at least LDI = 1 (i.e. one landrace) and *H*≥0.8933 (i.e. the median of the *H* data distribution obtained) were admitted to the following prioritization level (Fig 1, left; Table 1), so as not to exclude any area where landraces were present.

Concerning Criterion 2 (PWS), by using the EUAP-N2N overlapped layer, only quadrants with an area covered by protected areas greater than or equal to 80 km^2^ (20% of quadrant surface area) were taken into consideration (Fig 1, left; Table 1) so as not to exclude any protected area of significant surface in a quadrant.

Concerning Criterion 3 (AED) only the quadrants with an overall area covered by Levels 2, 3, 4 and 5 of CLC (2006) greater than or equal to 360 km^2^ (i.e. 90% of quadrant area covered by agricultural land, forests and semi-natural areas, wetlands and water surfaces) were admitted to the following sieve (Fig 1, left; Table 1). This made it possible to include the highest land use diversity per unit of area.

In the AS an arcsine transformation of index values [81] was initially applied in order to stabilize variances and normalize proportional data. The threshold values were defined as above, but the sum of these (normalized) values was taken as a general threshold (137.9, Table 1). Only those quadrants whose total index value was greater than or equal to the threshold were selected as MAPAs, irrespectively of the score achieved for each singular index (Fig 1, right; Table 1).

## Results

To facilitate the reader, data will be presented making prevalent reference to administrative subdivisions of the sample area (i.e. the Italian Regions) (see “S1 Fig”).

### Landrace diversity (LRD) in the sample area

Of the 2,365 landraces inventoried across Italy [32], the highest landrace numbers were recorded in Umbria (378), Calabria (288), Sicily (251), Basilicata (212) and Campania (203). These Italian Regions accounted for more than 50% of total recorded landraces. No landrace data were obtained from the Puglia Region. The most frequently found landraces are fruit trees (73% of the total of recorded landraces: apples, pears, plums, grapes, olive and other tree species, in order of frequency), followed by herbaceous plants (27%: grain legumes, vegetables, cereals and forages, in order of frequency) as also previously recorded [64].

**Fig 2:**
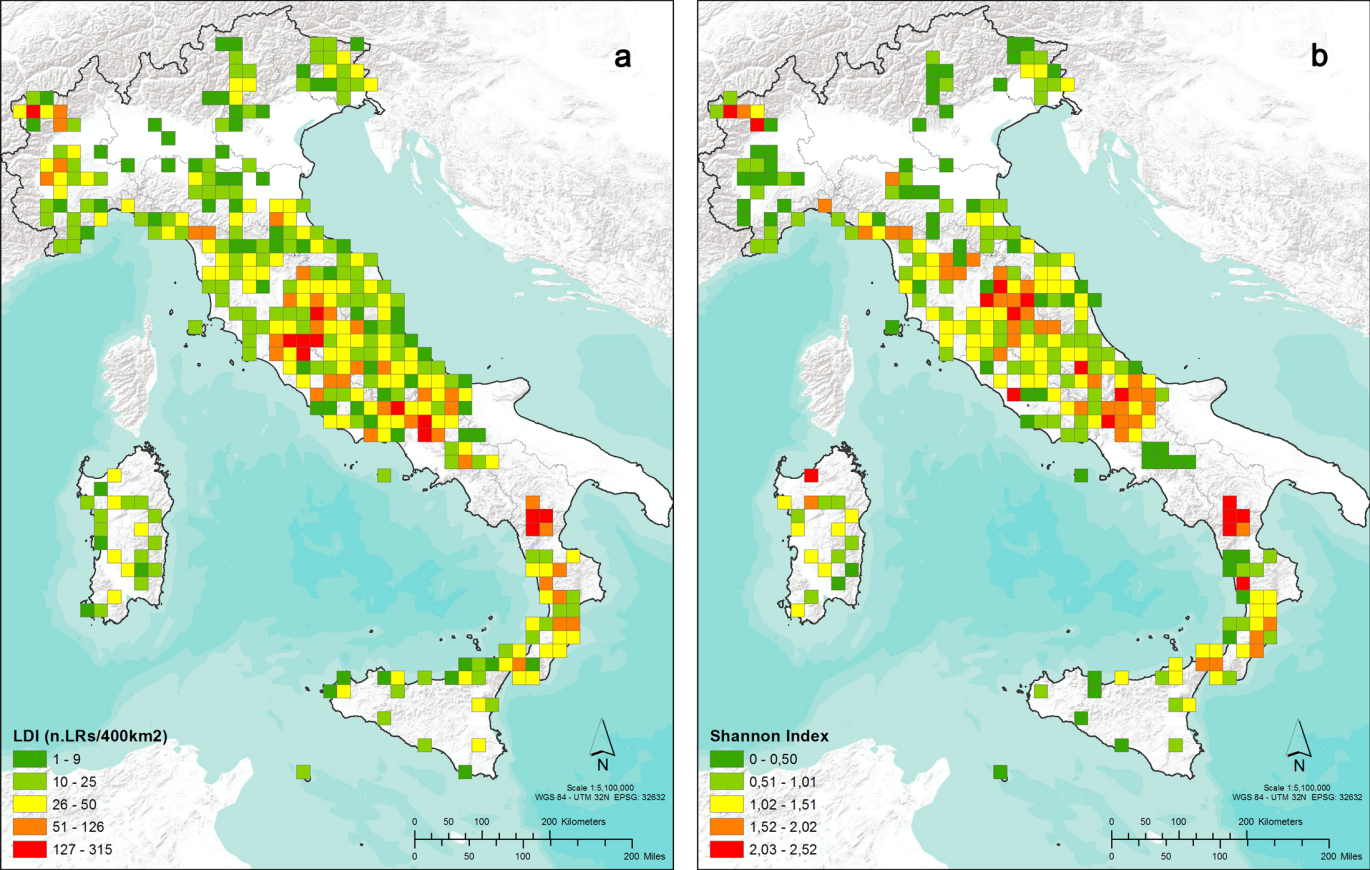
Landrace density index (a) and Shannon index (b) relative to landraces recorded in Italy.

The analysis of landrace diversity estimated through LDI and H' shows that the highest values of LDI were found in quadrants located in Umbria, Basilicata and Lazio, the Regions that have the highest number of landraces (LDI mean value per Region = 27, 26 and 23, respectively, see also Fig 2A), while the highest values of H' were detected in Basilicata, Molise and Umbria, the Regions that have the highest number of species cultivated as landraces (*H'* mean value per Region = 1.98, 1.60 and 1.58, respectively, see also Fig 2B). However, fairly good values of diversity were also detected in other Regions of Northern (Valle D’Aosta), Central (Toscana and Abruzzo) and Southern Italy (Calabria) [64].

Apart from a few exceptions, the areas richest in number of landraces and in different crops cultivated as landraces were located inland in hilly and mountainous areas of the Apennine chain that are characterized by high ecological diversity. The Apennines is a land system that preserves, and in some case enhances, the biological diversity threatened by changes in land use and by diffuse abandonment [82].

### Presence of wild species (PWS) in the sample area

**Fig 3:**
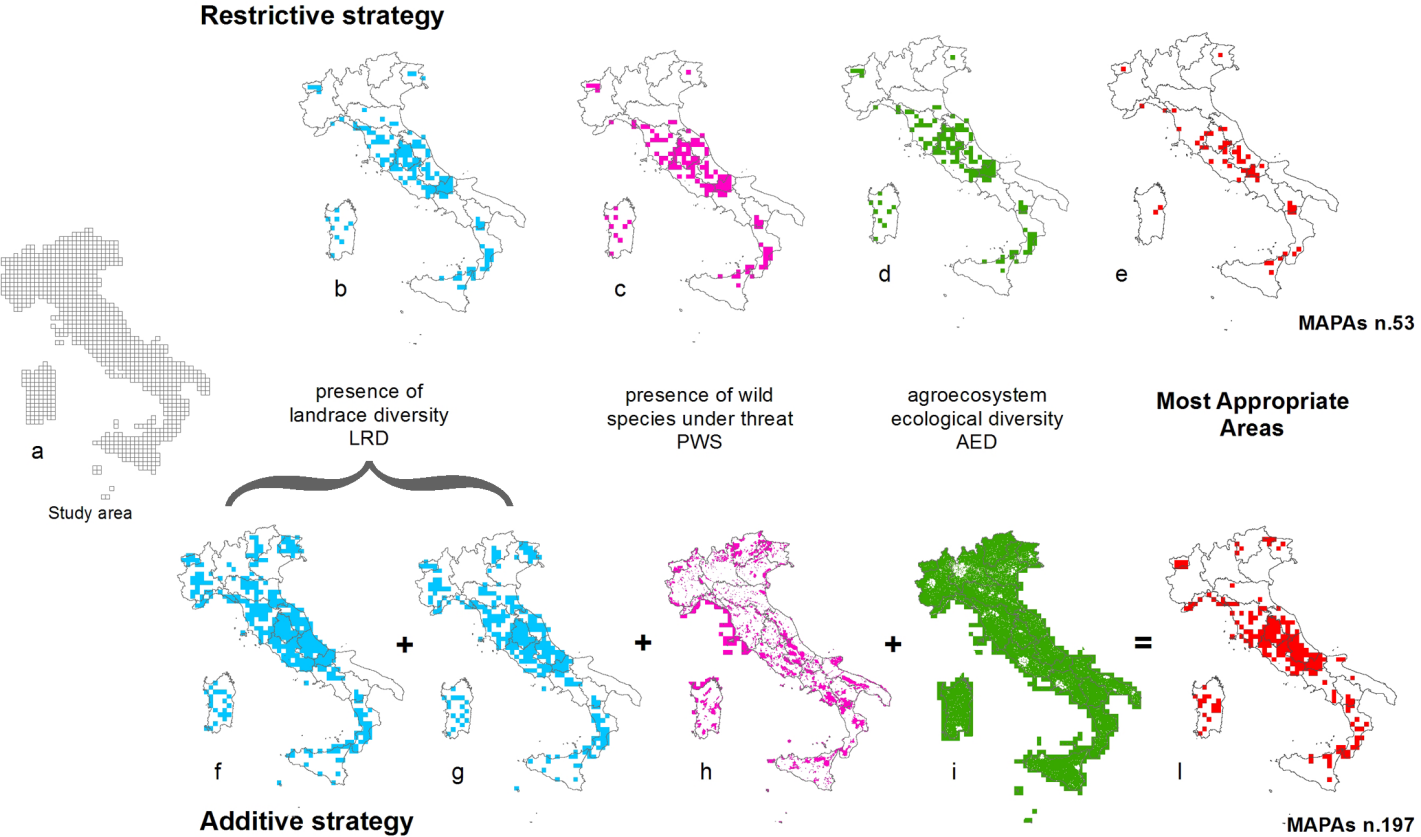
Quadrants progressively taken into consideration and finally identified as MAPAs by applying the two strategies. Quadrants with landrace diversity (LRD) (b, f, g): blue. Quadrants with wild species under threat (PWS) (c, h): purple. Quadrants with agro-ecosystem ecological diversity (AED) (d, i): green. Finally identified MAPAs quadrants (e, l): red.

Taking the percentage of territory covered by protected areas as a proxy of wild species, the PWS highest values are recorded in Abruzzo (37%), Campania (36%), Valle D’Aosta (30%), Lazio (28%), Liguria, Calabria, Molise (27%) and Trentino Alto Adige (26%). The Regions with the lowest value are Emilia Romagna (12%) and Toscana (15%). Most of these protected areas are located in hill and mountain landscapes in Italy (see Fig 3).

### Agro-ecosystem ecological diversity (AED) in the sample area

Taking the percentage of different uses of a certain territory as a proxy of agro-ecosystem diversity and considering only the most represented categories (i.e. Levels 2 and 3), the highest percentage of the land use category 2 (Agricultural areas) is recorded in Puglia (84%), followed by Sicilia, Emilia Romagna, Marche and Molise (69, 68, 65 and 63%, respectively) and that of the category 3 (Forests and semi-natural areas) in Valle d’Aosta followed by Trentino Alto Adige, Liguria and Abruzzo (90, 83, 87 and 50%, respectively).

### MAPAs identified with the RS and AS in the sample area

Using the *RS*, 53 MAPAs were identified (Fig 3B–3E), mainly located in the Regions of Lazio, Abruzzo, Molise, Umbria and Basilicata. Their location is directly proportional to the high LRD of these Regions.

The *AS* identified a larger number of MAPAs than identified with the RS, i.e. 197 (Fig 3F–3I), obviously all the MAPAs defined by means of the RS were included.

The main difference between the two strategies lies in the fact that the AS established a number of MAPAs in Regions such as Veneto and Emilia Romagna, where no MAPAs were identified by RS (Fig 3) and in Regions where, although no data on landrace were recorded, many protected areas and large agro-ecosystem ecological diversity exist (i.e. Puglia).

The MAPAs identified by both strategies are mainly located in the Mediterranean bio-geographical region (80% and 73% of those identified with the RS and AS, respectively) and could be considered as sub-hotspots in a region which is one of the most important biodiversity hotspots in the world.

**Fig 4:**
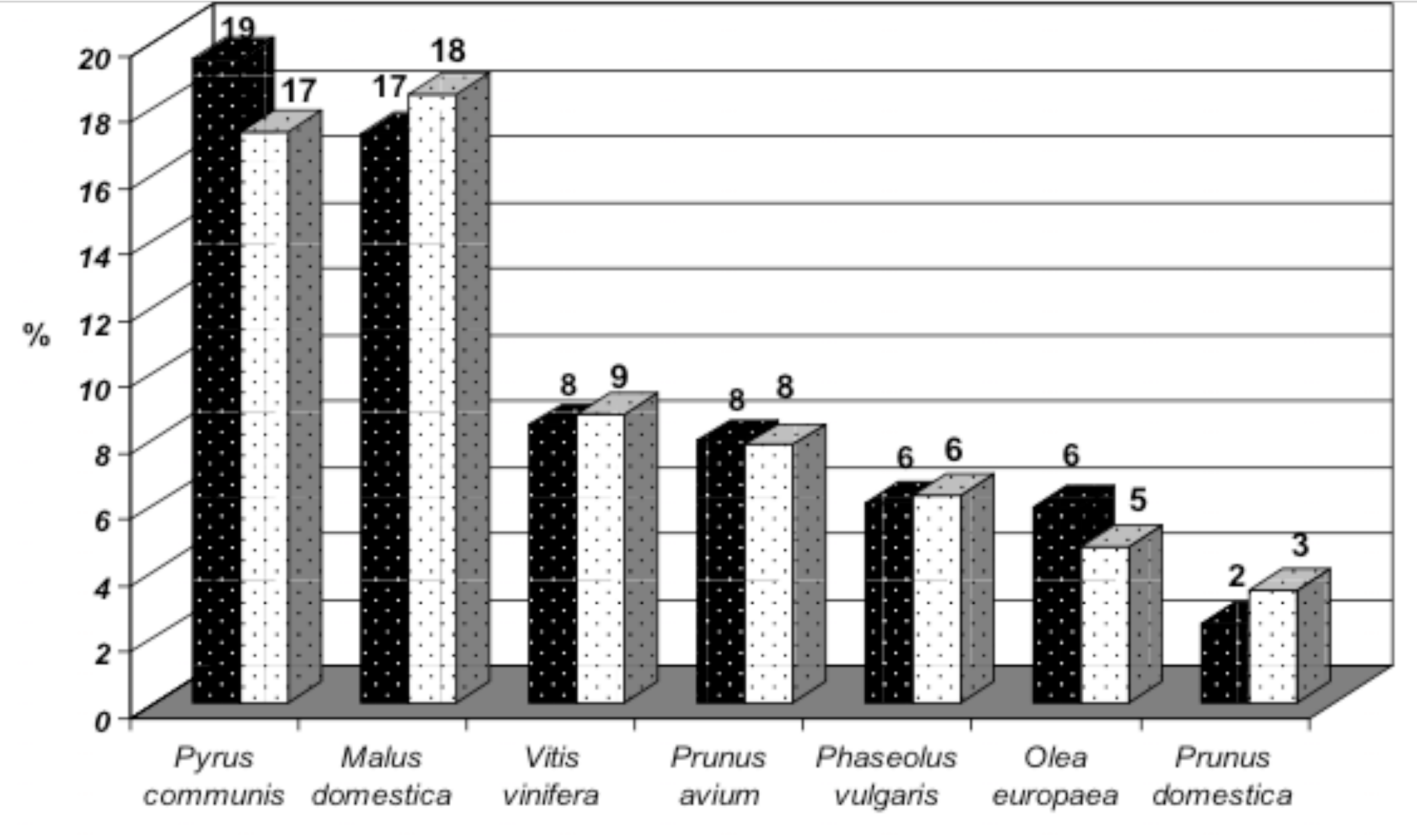
Percentages of the most frequently recorded species cultivated as landraces in the MAPAs identified through the RS (black columns) and the AS (white columns) prioritization strategies.

By analyzing the presence of species cultivated as landraces in the MAPAs it was found that both strategies indicated the prevalence of the same species: *Pyrus communis* L., *Malus domestica* Borkh., *Vitis vinifera* L., *Prunus avium* L., *Phaseolus vulgaris* L., *Olea europaea* L. and *Prunus domestica* L. (Fig 4). Landraces of these crops are mostly found on hill sites (over 400 m asl), *V*. *vinifera* and *Prunus spp* landraces are mostly recorded in the northern part of the country (at an average latitude of 42.89° N and 41.81° N, respectively) while *M*. *domestica* and *P*. *communis* landraces in the southern part (at an average latitude 41.79° N and 40.96° N, respectively).

Table 2 shows the range values of each index in the MAPAs identified with the two prioritization strategies. While the maximum values in the areas identified as MAPAs with both strategies were quite similar, differences were found for the minimum values of PWS and AED of some areas. Being substantially independent by a threshold for each parameter, or, in other words, when carried out without any sieve, the AS identified at one extreme MAPAs with values of PWS and AED lower than those identified by the RS. As such the AS appears to be less efficient than the RS in capturing total agro-biodiversity.

**Table 2:** Value range of the indices detected in the 53 and 197 MAPAs identified with the RS and the AS prioritization strategies, respectively.

Criterion	Index	RS MAPAs^a^ *value range*		AS MAPAs^b^ *value range*	
		*min*	*max*	*min*	*max*
C1 –LRD^c^	LDI^d^	3	315	0	315
	H’^e^	0.895	2.525	0	2.525
C2 –PWS^f^	EUAP-N2K^g^	20.88%	90.79%	0.06%	99.29%
C3 –AED^h^	*CLC–L 2+3+4+5* ^i^	90.96%	99.81%	53.56%	100%

^a^ Restrictive Strategy for identify Most Appropriate Areas

^b^ Additive Strategy for identify Most Appropriate Areas

^c^ Criterion 1—Landrace diversity

^d^ Landrace Density Index

^e^ Shannon Index

^f^ Criterion 2—Presence of wild species under threat

^g^ Elenco Ufficiale delle Aree Naturali Protette (Ministero dell'Ambiente, della Tutela del Territorio e del Mare) and Natura 2000 Network (European Commission)

^h^ Agro-ecosystem Ecological Diversity

^i^ Corine Land Cover, level 2,3,4,5.

Of course the choice of different thresholds from those used would have yielded different results. It has to be noted, however, that the main intent of this paper was to propose methods for identifying and prioritizing agro-biodiversity hotspots for *in situ* conservation of plant genetic resources and test the feasibility of such approaches at regional level.

## Discussion

International treaties and conventions to which many countries are signatories require actions in order to promote agro-biodiversity conservation [1,11,24–26]. In particular the CBD 1992 [1] defines *in situ* conservation as ” the conservation of ecosystems and natural habitats and the maintenance and recovery of viable populations of species in their natural surroundings and, in the case of domesticated or cultivated species, in the surroundings where they have developed their distinctive properties” as such clearly including cultivated forms as landraces.

While several spatial and ecogeographic approaches for selecting areas which deserve active conservation have been proposed, focusing on particular components of agro-ecosystems [41,52], this paper reports a first attempt to set a methodological approach to identify agro-biodiversity rich areas by applying a holistic view (i.e. globally and contextually considering the diversity within species, among species and ecosystems) and considering the maintenance of genetic resources as a service to agro-ecosystems [13].

Our focus was on crop genetic resources, since several international documents [11,24–26] have highlighted their continuous loss and have stressed the need for preserving this component of biodiversity to feed the human world in the future, when considering the present climate change scenario.

It is not always easy to find the right link between diversity indices and biodiversity measures [43] and the really important issue is not only to measure, but also to establish, indices which allow monitoring over time [83,84].

Concerning the criteria used to assess agro-biodiversity components and ways to apply them, we used those that have already been discussed by a large number of experts and tested on a case study [64]. Of course, additional criteria (and tools to assess them on a geographic basis, as in our approach) could have been additionally taken into account to estimate the biodiversity level of a certain area. Among them, the phytoclimatology of the sites (especially considering that in some areas diversity is at risk due to climate change [85]) or the presence of Prime Butterfly Areas [47] or even, extending the focus, the diversity of local animal breeds and of the human socio-cultural context could have been considered, provided relative data were available. However, we simply aimed to set methods for prioritizing areas of interest, and not to find out which areas are actually the most agro-biodiverse. Moreover, in our opinion any other criterion concerning agro-biodiversity assessment can be added to the strategies developed here without affecting their functionality.

As for the methods to assess each component, we relied on objective tools that are available for large territories (i.e. official inventories, location of protected areas and CLC) and can be continuously updated, thus also allowing the development of monitoring actions.

The first criteria applied was LRD which was assessed by considering both number and diversity of landraces per quadrant.

The richness in wild species number is often used to identify areas of high biodiversity value and to develop biodiversity conservation strategies [86–90]. Here we extended the richness concept to crop species cultivated as landraces and took into account the relative presence of landraces belonging to a certain crop using ‘*H’* for quadrants where landraces were present. Of course, in order to work out an LRD, basic information on landrace existence in the area is needed. At present, inventories for extant landraces (i.e. landraces that still exist *in situ*) that are complete with geographic information have been compiled only for Finland and Italy [32,35] while there are fragmentary inventories in other European countries, (see various authors) [27], and, to the best of our knowledge, do not exist at all in other regions of the world. However, Descriptors and tools to compile landrace inventories have recently been made available by EC FP7 PGR Secure project [91,92] which will make it possible to compile such inventories easily in all countries of the world and, consequently, to apply LRD as a criterion to assess agro-biodiversity and plan conservation activities in the future.

It should also be noted that to assess LRD we only used presence and diversity of landraces per quadrant, while indeed landraces have other types of features which could have been taken into account and assessed in some ways. Landraces contribute differently to people’s income (for family use alone, or destined for the market), allow conservation and development of crop diversity in different ways in response to changing conditions and the diversification of products from agriculture (food, feed, seed and ecosystem benefits and services). All these different features and the ways they are implemented depend on crop, location, use, market and the existing socio-economic and cultural context. Finally, landraces run different risks of extinction as they are covered by different types of protection measures and evaluation schemes [27,63,93–95]. How much diversity exists among landraces relative to all these features? How do we estimate the multiple and associated characteristics of a landrace? This is not simple and this difficulty has not yet been overcome and so has hampered us in carrying out such an assessment.

To apply Criterion 2, Presence of wild species, we had no other possibility but to proxy estimate this component considering the areas covered by protected areas. As mentioned above, protected areas, beside protecting different forms of life, can also provide food, shelter and disperser corridors for useful insects (such as pollinators and pest predators) which are important for the health of agricultural systems, in addition to numerous other ecosystem services. As such they were considered a reliable proxy.

Being mainly concerned with the species that are most useful in agriculture for food security in a scenario of climate change and unpredictability (i.e. plant genetic resources), it would have been useful to include precise geographic location data relative to the crop wild relatives. However, in Italy at present, while information on the crop wild relatives of the main crops is available for protected areas [72], it is not fully available for other areas. This also occurs in other countries [96,97]. Crop wild relative lists are currently being developed, taking advantage of genebank holding records which can serve to localize their populations later, for Italy [36]; Venezuela [98]; Spain [99]; USA [100]; Portugal [101]; United Kingdom [102] andFinland [103].

Agro-ecosystem ecological diversity is a major driver of biodiversity and is an important parameter in defining conservation strategies [74,104,105]. To apply this criterion, the Corine Land Cover was used which is qualified for use in such an assessment for the whole of Europe. This dataset of land-use includes time-series data (available since 1990) and consequently also permits the evaluation of the transformation of natural ecosystems to semi-natural, agricultural or artificial systems which is the major cause of biodiversity loss. The analysis of land use changes is a key component to assess the loss of biodiversity worldwide [106,107].

Although different systems are applied in some countries to assess land use, such as remote sensing data [108], systems similar to CLC in the USA (i.e. the National Land Cover Database) or Australia [109], the method used here can be useful in other geographical situations as well.

As regards the strategies developed, indeed the RS identifies a lower number of MAPAs than the AS, but, when few resources are available, it can be useful to address resources for conservation specifically where agro-biodiversity is highest.

The AS, although being less efficient than the RS, has the advantage of identifying areas which lack basic data for some of the agro-biodiversity components taken into account (e.g. for those areas where no data on landrace existence are recorded) which, in turn, offers the possibility of taking action anyway in the form of gathering additional data for more stringent actions.

The AS takes advantage of the maximum range of recorded presence of protected areas, but, to a certain extent, also considers the presence of landraces. On this point, it should also be noted that many landraces are still maintained on-farm in protected areas [55] and that landraces are continuously being detected (Negri pers. comm.). Protected areas are mainly confined to the most marginal areas of a country (like mountains and valleys among mountains) where soil moisture, exposure to sun and wind, temperature, and evapo-transpiration rates are each highly variable giving rise to a myriad of different ecological niches. In these landscapes modern varieties, usually bred for highly uniform environments and based on a single genotype, do not perform as well as landraces. Landraces, contrary to modern varieties, have become adapted to the difficult climatic and pedological conditions of these areas, as diverse and plastic populations each fitting into a specific environmental niche, through centuries or millennia of natural and human selection in cultivation and isolation [93] which explains why they are most widespread in these areas.

From the point of view of efficiency in identifying species cultivated as landraces, both strategies are almost similar. Obviously, long-living species (i.e. trees) and species of which the seed is the product of farming (i.e. *Phaseolus*) are the most easily maintained as landraces in the area we used to develop this methodological approach.

The choice of thresholds obviously conditions the results that can be obtained (i.e. the number of identified MAPAs). Of course, when a similar study is carried out in a different context, thresholds should be defined according to the particular characteristics of the targeted areas (for which more or less data may be available, which may have greater or lesser ecological diversity etc), the administrative context where the conservation strategy is to be applied (National, Regional, Municipal etc), the local political orientation and the resources available for setting conservation areas.

This study also shows that geographic information data available on a large (i.e. European) scale such as the Corine provide an effective tool to analyse agro-biodiversity. Modern tools like GIS applications represent a key element in mapping species distribution, creating distribution models and defining an eco-geographical land characterization which also takes into account crop genetics [45, 51–54] so as to lead to efficient conservation planning.

## Conclusions

The identification of biodiversity hotspots to be conserved with priority allows for the highest level of diversity at the lowest cost [4]. Considering agro-biodiversity, in addition to habitats and wild species, hotspots must include genetic resources and specific landraces and crop wild relatives which are the most threatened. As far as we know this is the first methodological approach to identify agro-biodiversity hotspots (MAPAs) in Europe by means of a prioritization process. The identified MAPAs can be recommended to National or Regional authorities as areas in which to set up or enhance political and economic actions in favour of agro-biodiversity conservation with priority, also relying on the agro-environmental measures already foreseen by the Common Agricultural Policies in the case of the European Union [110].

As mentioned in the “Concept for on-farm conservation and management”, produced by the European Cooperative Program for Plant Genetic Resources [94], the creation of a network of MAPAs at European level would also facilitate the development of *in situ* conservation plans and their implementation, the attraction of funds for research on diversity and for monitoring trends in response to climate change and human activities and, finally, the identification of unique *in situ* plant genetic resources for *ex situ* back-up conservation.

Priority sites designated as hotspots of diversity would also suit the Man and the Biosphere-Plan–Biosphere Reserves Strategy of Sevilla [111]. Biosphere Reserves are in fact intended to fulfill three complementary functions: “a conservation function” (to preserve genetic resources, species, ecosystems and landscapes), “a development function” (to foster sustainable economic and human development) and “a logistic support function” (to support demonstration projects, environmental education and training, and research and monitoring related to local, national and global issues of conservation and sustainable development). In addition, when including landraces, MAPAs could also be included among the FAO Globally Important Agricultural Heritage Systems.

The criteria used and methodology presented here could be easily applied on a European level and anywhere else similar data and geographic information systems exist. However, for most of the indicators used here, data are frequently unavailable or insufficient worldwide. At the European level in particular, more data on occurrence and location of landraces and crop wild relatives are currently needed, which lays a claim for urgent actions.

## Supporting information

S1 Fig Administrative subdivision of the Italian regions. (TIF)

S1 Table Details of 995 quadrants, with information on georeferentation, LRD, PWS and AED indices. (DOCX)

S2 Table Italian corine land cover class. (DOCX)
