# Supplementary figures and images for: A methodological approach to identify agro-biodiversity hotspots for priority in situ conservation of plant genetic resources
Source: PLoS One. 2018 Jun 1;13(6):e0197709. doi: 10.1371/journal.pone.0197709 (PMC5983459; doi:10.1371/journal.pone.0197709)

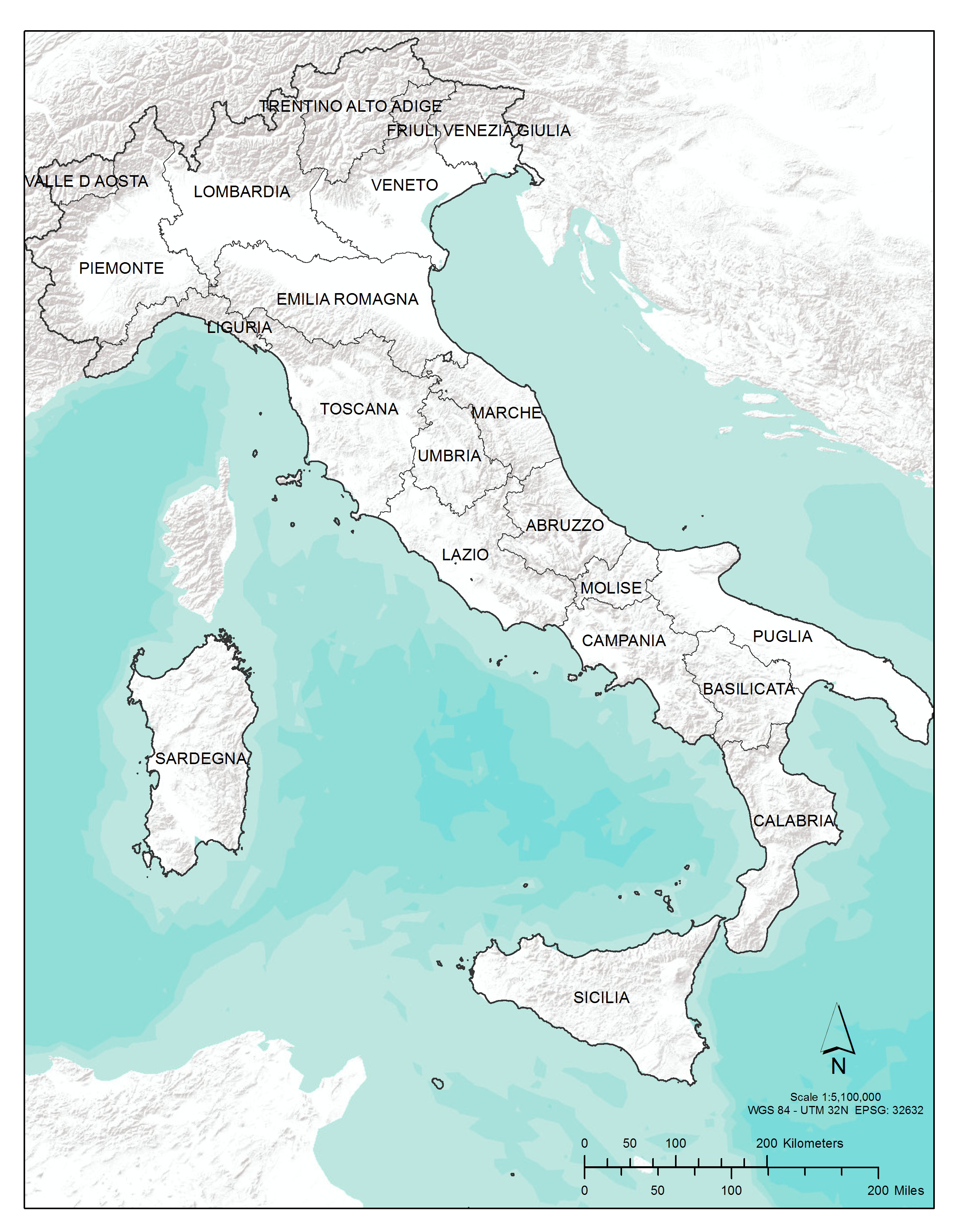

Supplement: S1 Fig — (TIF) [file pone.0197709.s001.tif]
