# Supplementary material for: A methodological approach to identify agro-biodiversity hotspots for priority in situ conservation of plant genetic resources
Source: PLoS One. 2018 Jun 1;13(6):e0197709. doi: 10.1371/journal.pone.0197709 (PMC5983459; doi:10.1371/journal.pone.0197709)
Supplement: S1 Table — (DOCX) [file pone.0197709.s002.docx]

S1 Table. Details of 995 quadrants, with information on georeferentation, LRD, PWS and AED indices.

The table shows the 995 quadrants which cover Italy, each one identified by coordinates (decimal degrees format) and the single value for each indexes.

In the field called Id quadrants, were reported with three *** the quadrants identified in both strategies and with two ** the quadrants identified in AS.

| Id quadrants | Latitude DD | Longitude DD | LRD | | PWS | AED |
| --- | --- | --- | --- | --- | --- | --- |
|  |  |  | **LDI** | **Shannon** | **EUAP-N2K** | **CLC** |
| 0 | 35.58658 | 12.33757 | 0 | not available | 0.03063 | 0.84215 |
| 1 | 35.58027 | 12.55792 | 0 | not available | 0.18872 | 0.99237 |
| 2 | 35.93342 | 12.79528 | 0 | not available | 0.08657 | 0.9989 |
| 3 | 36.67848 | 11.93670 | 0 | not available | 0.10138 | 0.99504 |
| 4 | 36.67275 | 12.16022 | 0 | not available | 0.01894 | 0.98958 |
| 5 | 36.56039 | 15.05829 | 0 | not available | 0.00017 | 0.99984 |
| 6 | 36.85853 | 11.94358 | 2 | 0 | 0.24499 | 0.9838 |
| 7 | 36.85277 | 12.16762 | 0 | not available | 0.02995 | 0.99844 |
| 8 | 36.77203 | 14.40359 | 0 | not available | 0.01034 | 0.99481 |
| 9 | 36.76166 | 14.62665 | 1 | not available | 0.01366 | 0.9733 |
| 10 | 36.75088 | 14.84959 | 0 | not available | 0.02662 | 0.93972 |
| 11 | 36.73967 | 15.07241 | 0 | not available | 0.1644 | 0.98209 |
| 12 | 36.96154 | 14.19259 | 0 | not available | 0.02685 | 0.99865 |
| 13 | 36.95152 | 14.41628 | 0 | not available | 0.18443 | 0.97642 |
| 14 | 36.94108 | 14.63985 | 0 | not available | 0.023 | 0.93395 |
| 15 | 36.93023 | 14.86331 | 0 | not available | 0.08339 | 0.98629 |
| 16 | 36.91895 | 15.08664 | 0 | not available | 0.1922 | 0.96196 |
| 17 | 36.90726 | 15.30985 | 0 | not available | 0.02018 | 0.98881 |
| 18 | 37.16881 | 13.53161 | 0 | not available | not available | 0.99361 |
| 19 | 37.15999 | 13.75613 | 0 | not available | 0.02464 | 0.98776 |
| 20 | 37.15075 | 13.98055 | 2 | 0 | 0.02389 | 0.97163 |
| 21 | 37.14109 | 14.20487 | 0 | not available | 0.06823 | 0.94704 |
| 22 | 37.13100 | 14.42908 | 0 | not available | 0.46865 | 0.98933 |
| 23 | 37.12049 | 14.65318 | 0 | not available | 0.02091 | 0.98947 |
| 24** | 37.10957 | 14.87716 | 7 | 0.41012 | 0.2141 | 0.98944 |
| 25 | 37.09822 | 15.10101 | 0 | not available | 0.19746 | 0.94348 |
| 26 | 37.08645 | 15.32474 | 0 | not available | 0.05547 | 0.92747 |
| 27 | 37.35697 | 13.31726 | 0 | not available | 0.03681 | 0.99172 |
| 28 | 37.34852 | 13.54241 | 0 | not available | 0.00936 | 0.92397 |
| 29 | 37.33965 | 13.76746 | 0 | not available | not available | 0.96981 |
| 30 | 37.33035 | 13.99242 | 0 | not available | 0.00356 | 0.98008 |
| 31 | 37.32062 | 14.21726 | 0 | not available | 0.01646 | 0.98953 |
| 32 | 37.31047 | 14.44200 | 0 | not available | 0.00249 | 0.98076 |
| 33 | 37.29989 | 14.66662 | 0 | not available | not available | 0.98192 |
| 34 | 37.28890 | 14.89112 | 0 | not available | 0.02851 | 0.97336 |
| 35 | 37.27747 | 15.11550 | 0 | not available | 0.08315 | 0.9291 |
| 36 | 37.26563 | 15.33975 | 0 | not available | 0.00037 | 0.9898 |
| 37 | 37.55965 | 12.65002 | 0 | not available | 0.04693 | 0.97183 |
| 38 | 37.55244 | 12.87597 | 0 | not available | 0.00991 | 0.97264 |
| 39 | 37.54480 | 13.10184 | 0 | not available | 0.19901 | 0.9792 |
| 40 | 37.53673 | 13.32762 | 2 | 0 | 0.1075 | 0.98407 |
| 41 | 37.52823 | 13.55331 | 0 | not available | 0.09621 | 0.99411 |
| 42 | 37.51929 | 13.77890 | 0 | not available | 0.01213 | 0.98023 |
| 43 | 37.50993 | 14.00438 | 0 | not available | 0.0335 | 0.96297 |
| 44 | 37.50014 | 14.22976 | 0 | not available | 0.10977 | 0.98028 |
| 45 | 37.48993 | 14.45503 | 0 | not available | 0.15721 | 0.99013 |
| 46 | 37.47928 | 14.68019 | 0 | not available | 0.01602 | 0.99448 |
| 47 | 37.46822 | 14.90522 | 0 | not available | 0.01591 | 0.94478 |
| 48 | 37.45672 | 15.13013 | 0 | not available | 0.09344 | 0.82766 |
| 49 | 37.74638 | 12.43228 | 0 | not available | 0.03084 | 0.92196 |
| 50 | 37.73956 | 12.65885 | 0 | not available | 0.09003 | 0.96612 |
| 51 | 37.73230 | 12.88535 | 0 | not available | 0.01677 | 0.95215 |
| 52 | 37.72461 | 13.11175 | 0 | not available | 0.14694 | 0.98302 |
| 53 | 37.71648 | 13.33807 | 0 | not available | 0.59879 | 0.98973 |
| 54 | 37.70792 | 13.56430 | 0 | not available | 0.35218 | 0.98111 |
| 55 | 37.69893 | 13.79043 | 0 | not available | 0.00548 | 0.99297 |
| 56 | 37.68951 | 14.01646 | 0 | not available | 0.01298 | 0.99601 |
| 57 | 37.67966 | 14.24238 | 0 | not available | 0.11482 | 0.99646 |
| 58 | 37.66938 | 14.46818 | 0 | not available | 0.05269 | 0.9769 |
| 59 | 37.65867 | 14.69387 | 0 | not available | 0.11235 | 0.99271 |
| 60*** | 37.64753 | 14.91944 | 10 | 0.94335 | 0.48161 | 0.90962 |
| 61 | 37.63596 | 15.14488 | 3 | 1.09861 | 0.17328 | 0.80723 |
| 62 | 37.93874 | 11.98618 | 0 | not available | 0.29601 | 0.99989 |
| 63 | 37.93275 | 12.21344 | 0 | not available | 0.82851 | 0.99917 |
| 64 | 37.92632 | 12.44064 | 1 | not available | 0.39404 | 0.94208 |
| 65 | 37.91945 | 12.66776 | 7 | 0.79631 | 0.03891 | 0.9849 |
| 66 | 37.91214 | 12.89480 | 0 | not available | 0.07565 | 0.96821 |
| 67 | 37.90440 | 13.12176 | 0 | not available | 0.01378 | 0.98796 |
| 68 | 37.89623 | 13.34862 | 0 | not available | 0.35462 | 0.98866 |
| 69 | 37.88761 | 13.57540 | 4 | 0 | 0.10341 | 0.98744 |
| 70 | 37.87857 | 13.80207 | 0 | not available | 0.22064 | 0.98402 |
| 71** | 37.86908 | 14.02864 | 0 | not available | 0.71777 | 0.98244 |
| 72 | 37.85917 | 14.25510 | 0 | not available | 0.19281 | 0.99125 |
| 73 | 37.84882 | 14.48145 | 0 | not available | 0.56267 | 0.99419 |
| 74 | 37.83804 | 14.70768 | 0 | not available | 0.39214 | 0.9915 |
| 75** | 37.82683 | 14.93378 | 0 | not available | 0.7578 | 0.97764 |
| 76 | 37.81518 | 15.15976 | 0 | not available | 0.33972 | 0.94623 |
| 77 | 37.80311 | 15.38561 | 0 | not available | 0.01289 | 0.99266 |
| 78 | 38.10625 | 12.44907 | 0 | not available | 0.02871 | 0.97991 |
| 79 | 38.09934 | 12.67675 | 1 | not available | 0.2744 | 0.93954 |
| 80 | 38.09198 | 12.90434 | 0 | not available | 0.11318 | 0.96866 |
| 81 | 38.08419 | 13.13185 | 0 | not available | 0.28994 | 0.88176 |
| 82 | 38.07596 | 13.35927 | 1 | not available | 0.16977 | 0.78322 |
| 83 | 38.06729 | 13.58659 | 6 | 0 | 0.06475 | 0.95391 |
| 84 | 38.05819 | 13.81381 | 0 | not available | 0.00129 | 0.97693 |
| 85** | 38.04865 | 14.04093 | 3 | 1.09861 | 0.16699 | 0.98233 |
| 86 | 38.03867 | 14.26794 | 0 | not available | 0.06861 | 0.98869 |
| 87 | 38.02825 | 14.49483 | 1 | not available | 0.42504 | 0.99175 |
| 88*** | 38.01740 | 14.72161 | 8 | 0.97431 | 0.54791 | 0.96778 |
| 89*** | 38.00611 | 14.94826 | 4 | 1.03972 | 0.3127 | 0.98931 |
| 90 | 37.99440 | 15.17478 | 0 | not available | 0.31902 | 0.9937 |
| 91 | 37.98224 | 15.40116 | 0 | not available | 0.11464 | 0.97816 |
| 92** | 37.96966 | 15.62741 | 18 | 1.21489 | 0.02684 | 0.98491 |
| 93** | 37.95665 | 15.85352 | 18 | 0.84869 | 0.20414 | 0.99178 |
| 94 | 37.94320 | 16.07948 | 0 | not available | 0.072 | 0.99409 |
| 95 | 38.27921 | 12.68581 | 0 | not available | 0.00032 | 1 |
| 96 | 38.26397 | 13.14203 | 0 | not available | 0.01924 | 0.97017 |
| 97 | 38.25569 | 13.37000 | 0 | not available | 0.08387 | 0.95117 |
| 98 | 38.19675 | 14.73566 | 1 | not available | not available | 0.97953 |
| 99 | 38.18539 | 14.96286 | 3 | 1.09861 | 0.01447 | 0.97424 |
| 100 | 38.17360 | 15.18993 | 1 | not available | 0.00788 | 0.9206 |
| 101*** | 38.16137 | 15.41686 | 13 | 1.52471 | 0.40629 | 0.92604 |
| 102** | 38.14871 | 15.64365 | 33 | 2.01684 | 0.24935 | 0.89125 |
| 103** | 38.13561 | 15.87030 | 1 | not available | 0.79595 | 0.99643 |
| 104 | 38.12208 | 16.09680 | 0 | not available | 0.30573 | 0.98393 |
| 105 | 38.10811 | 16.32315 | 0 | not available | not available | 0.99932 |
| 106 | 38.36466 | 14.97759 | 0 | not available | 0.20196 | 0.99691 |
| 107 | 38.35279 | 15.20521 | 0 | not available | 0.00072 | 0.99991 |
| 108** | 38.34049 | 15.43270 | 5 | 1.33218 | 0.0741 | 0.99391 |
| 109*** | 38.32774 | 15.66004 | 13 | 1.26363 | 0.4766 | 0.971 |
| 110 | 38.31456 | 15.88724 | 0 | not available | 0.24655 | 0.97185 |
| 111*** | 38.30094 | 16.11428 | 10 | 1.22753 | 0.44907 | 0.98557 |
| 112** | 38.28689 | 16.34118 | 14 | 1.56915 | 0.02176 | 0.97529 |
| 113 | 38.27240 | 16.56791 | 0 | not available | not available | 0.99786 |
| 114 | 38.62351 | 13.16267 | 0 | not available | 0.24055 | 0.99928 |
| 115 | 38.57710 | 14.30714 | 0 | not available | 0.07282 | 0.99905 |
| 116 | 38.56649 | 14.53571 | 0 | not available | 0.10672 | 0.99999 |
| 117 | 38.55543 | 14.76414 | 0 | not available | 0.15736 | 0.99286 |
| 118 | 38.54392 | 14.99246 | 0 | not available | 0.23957 | 0.97845 |
| 119 | 38.49350 | 15.90432 | 2 | 0 | 0.00095 | 0.93869 |
| 120 | 38.47979 | 16.13192 | 0 | not available | 0.06824 | 0.97152 |
| 121*** | 38.46565 | 16.35936 | 13 | 1.95126 | 0.29096 | 0.99276 |
| 122 | 38.45107 | 16.58664 | 12 | 0.56609 | 0.01106 | 0.99336 |
| 123 | 38.87598 | 8.36127 | 0 | not available | 0.01286 | 0.99994 |
| 124 | 38.87707 | 8.66685 | 0 | not available | 0.1047 | 0.99974 |
| 125 | 38.87769 | 8.97253 | 0 | not available | 0.03116 | 0.99261 |
| 126 | 38.87787 | 9.27916 | 0 | not available | 0.00001 | 0.99717 |
| 127 | 38.80327 | 13.17312 | 0 | not available | 0.17706 | 0.99994 |
| 128 | 38.72317 | 15.00745 | 0 | not available | 0.07618 | 0.9982 |
| 129 | 38.71115 | 15.23619 | 0 | not available | 0.1099 | 0.99942 |
| 130 | 38.67242 | 15.92156 | 6 | 0.63651 | 0.0471 | 0.9712 |
| 131 | 38.65863 | 16.14972 | 3 | 0.63651 | 0.02066 | 0.94238 |
| 132** | 38.64440 | 16.37771 | 31 | 1.4623 | 0.17191 | 0.97086 |
| 133** | 38.62972 | 16.60554 | 23 | 1.54401 | 0.00061 | 0.98206 |
| 134 | 39.05620 | 8.34442 | 1 | not available | 0.15164 | 0.98847 |
| 135** | 39.05729 | 8.65585 | 5 | 1.33218 | 0.09091 | 0.97577 |
| 136 | 39.05792 | 8.96738 | 0 | not available | 0.40093 | 0.99502 |
| 137 | 39.05809 | 9.27987 | 0 | not available | 0.02778 | 0.96195 |
| 138 | 39.05707 | 9.90351 | 0 | not available | 0.23088 | 0.98756 |
| 139 | 39.05587 | 9.21491 | 0 | not available | 0.00995 | 0.98818 |
| 140 | 38.89031 | 15.25189 | 0 | not available | 0.04323 | 0.99767 |
| 141 | 38.83746 | 16.16767 | 0 | not available | 0.02878 | 0.97314 |
| 142 | 38.82313 | 16.39622 | 5 | 1.05492 | not available | 0.96951 |
| 143 | 38.80837 | 16.62462 | 4 | 1.03972 | 0.00375 | 0.95475 |
| 144 | 38.79316 | 16.85284 | 0 | not available | not available | 0.99999 |
| 145 | 39.23641 | 8.32743 | 0 | not available | 0.19826 | 0.95928 |
| 146 | 39.23751 | 8.64476 | 0 | not available | 0.0682 | 0.93696 |
| 147** | 39.23814 | 8.96219 | 6 | 0.86756 | 0.28654 | 0.99202 |
| 148 | 39.23832 | 9.28059 | 0 | not available | 0.20405 | 0.82841 |
| 149 | 39.23803 | 9.59862 | 0 | not available | 0.05795 | 0.89432 |
| 150 | 39.23728 | 9.91603 | 0 | not available | 0.26967 | 0.98403 |
| 151 | 39.23608 | 9.23333 | 0 | not available | 0.00414 | 0.99995 |
| 152 | 39.03024 | 15.95650 | 0 | not available | not available | 1 |
| 153 | 39.01627 | 16.18578 | 7 | 0 | 0.00592 | 0.96483 |
| 154** | 39.00186 | 16.41490 | 46 | 1.51181 | 0.00475 | 0.95846 |
| 155** | 38.98700 | 16.64386 | 4 | 1.03972 | 0.17828 | 0.9683 |
| 156 | 38.97169 | 16.87264 | 0 | not available | 0.18635 | 0.97761 |
| 157 | 38.95594 | 17.10124 | 0 | not available | 0.35572 | 0.97942 |
| 158 | 39.41662 | 8.31027 | 0 | not available | 0.18731 | 0.99381 |
| 159 | 39.41772 | 8.63356 | 0 | not available | 0.5316 | 0.98736 |
| 160 | 39.41836 | 8.95695 | 0 | not available | 0.09385 | 0.97994 |
| 161 | 39.41853 | 9.28131 | 0 | not available | not available | 0.94244 |
| 162 | 39.41825 | 9.60530 | 3 | 0 | 0.44044 | 0.98326 |
| 163 | 39.41749 | 9.92867 | 0 | not available | 0.44749 | 0.98937 |
| 164 | 39.41628 | 9.25192 | 0 | not available | 0.01643 | 0.99894 |
| 165 | 39.20913 | 15.97420 | 0 | not available | 0.00177 | 0.98502 |
| 166** | 39.19507 | 16.20405 | 26 | 2.24839 | 0.00094 | 0.9718 |
| 167 | 39.18056 | 16.43375 | 0 | not available | 0.16949 | 0.98621 |
| 168 | 39.16561 | 16.66327 | 0 | not available | 0.64158 | 0.98799 |
| 169** | 39.15021 | 16.89262 | 0 | not available | 0.68925 | 0.97975 |
| 170 | 39.13436 | 17.12179 | 0 | not available | 0.20231 | 0.96109 |
| 171 | 39.59681 | 8.29297 | 0 | not available | 0.01607 | 0.99987 |
| 172 | 39.59792 | 8.62226 | 0 | not available | 0.35513 | 0.97338 |
| 173 | 39.59857 | 8.95166 | 0 | not available | 0.02381 | 0.96941 |
| 174** | 39.59874 | 9.28204 | 11 | 1.16223 | 0.02127 | 0.97151 |
| 175 | 39.59846 | 9.61205 | 1 | not available | not available | 0.98761 |
| 176 | 39.59770 | 9.94142 | 2 | 0 | not available | 0.99525 |
| 177 | 39.59648 | 9.27069 | 0 | not available | 0.01562 | 0.99978 |
| 178 | 39.38801 | 15.99206 | 11 | 0 | 0.01031 | 0.98174 |
| 179 | 39.37386 | 16.22249 | 7 | 0.68291 | 0.01249 | 0.91313 |
| 180** | 39.35926 | 16.45276 | 26 | 0.64418 | 0.55284 | 0.98748 |
| 181 | 39.34421 | 16.68285 | 0 | not available | 0.58433 | 0.98864 |
| 182 | 39.32871 | 16.91277 | 0 | not available | 0.57175 | 0.99063 |
| 183 | 39.31277 | 17.14251 | 0 | not available | 0.07059 | 0.98106 |
| 184 | 39.77701 | 8.27550 | 0 | not available | 0.12375 | 0.99998 |
| 185 | 39.77812 | 8.61086 | 0 | not available | 0.19021 | 0.97548 |
| 186** | 39.77877 | 8.94632 | 6 | 1.0114 | 0.03229 | 0.9806 |
| 187 | 39.77895 | 9.28277 | 0 | not available | 0.12802 | 0.97475 |
| 188 | 39.77866 | 9.61885 | 2 | 0.69315 | 0.09591 | 0.98591 |
| 189 | 39.77790 | 9.95429 | 0 | not available | 0.08697 | 0.99102 |
| 190 | 39.77667 | 9.28961 | 0 | not available | 0.05767 | 0.99588 |
| 191 | 39.58065 | 15.77891 | 0 | not available | 0.0108 | 0.98661 |
| 192 | 39.56687 | 16.01008 | 3 | 0 | 0.0937 | 0.98414 |
| 193 | 39.55264 | 16.24109 | 2 | 0 | 0.01231 | 0.98058 |
| 194 | 39.53794 | 16.47194 | 0 | not available | 0.20028 | 0.98428 |
| 195 | 39.52280 | 16.70261 | 6 | 0.45056 | 0.12631 | 0.9821 |
| 196 | 39.50720 | 16.93311 | 0 | not available | 0.08864 | 0.98535 |
| 197 | 39.49115 | 17.16343 | 0 | not available | 0.0019 | 0.99992 |
| 198 | 39.95719 | 8.25788 | 0 | not available | 0.57972 | 0.99646 |
| 199 | 39.95832 | 8.59936 | 1 | not available | 0.214 | 0.93924 |
| 200 | 39.95897 | 8.94093 | 0 | not available | 0.05245 | 0.98785 |
| 201 | 39.95915 | 9.28352 | 0 | not available | 0.10806 | 0.98818 |
| 202** | 39.95886 | 9.62572 | 0 | not available | 0.73117 | 0.99328 |
| 203** | 39.95809 | 9.96728 | 2 | 0.69315 | 0.21689 | 0.98658 |
| 204 | 39.95685 | 9.30872 | 0 | not available | 0.07938 | 0.97541 |
| 205 | 39.75959 | 15.79650 | 0 | not available | 0.17657 | 0.96943 |
| 206** | 39.74572 | 16.02827 | 0 | not available | 0.79008 | 0.99453 |
| 207 | 39.73140 | 16.25986 | 0 | not available | 0.0269 | 0.97016 |
| 208 | 39.71661 | 16.49129 | 0 | not available | 0.01724 | 0.97091 |
| 209 | 39.70137 | 16.72255 | 0 | not available | 0.01223 | 0.99603 |
| 210 | 40.13738 | 8.24010 | 0 | not available | 0.00076 | 1 |
| 211** | 40.13851 | 8.58775 | 5 | 1.33218 | 0.09139 | 0.98425 |
| 212 | 40.13916 | 8.93550 | 0 | not available | 0.21805 | 0.98284 |
| 213 | 40.13934 | 9.28426 | 0 | not available | 0.14329 | 0.99169 |
| 214*** | 40.13905 | 9.63265 | 7 | 1.15374 | 0.34801 | 0.98406 |
| 215** | 40.13828 | 9.98038 | 0 | not available | 0.72611 | 0.99921 |
| 216 | 40.13703 | 9.32799 | 0 | not available | 0.309 | 0.99986 |
| 217 | 39.96505 | 15.34907 | 0 | not available | 0.31625 | 0.99418 |
| 218 | 39.95202 | 15.58174 | 0 | not available | 0.02296 | 0.99891 |
| 219 | 39.93852 | 15.81426 | 0 | not available | 0.36911 | 0.97896 |
| 220*** | 39.92457 | 16.04661 | 235 | 2.2991 | 0.85551 | 0.98888 |
| 221*** | 39.91015 | 16.27880 | 48 | 1.88927 | 0.90793 | 0.98797 |
| 222 | 39.89527 | 16.51082 | 0 | not available | 0.3461 | 0.98793 |
| 223 | 39.77833 | 18.12978 | 0 | not available | 0.15671 | 0.99539 |
| 224 | 39.75981 | 18.36025 | 0 | not available | 0.01754 | 0.98314 |
| 225 | 40.31755 | 8.22216 | 0 | not available | 0.10069 | 0.99987 |
| 226** | 40.31869 | 8.57604 | 2 | 0.69315 | 0.33109 | 0.98076 |
| 227** | 40.31935 | 8.93002 | 0 | not available | 0.6927 | 0.98163 |
| 228 | 40.31953 | 9.28502 | 0 | not available | 0.29377 | 0.97638 |
| 229 | 40.31924 | 9.63964 | 0 | not available | 0.0515 | 0.97009 |
| 230*** | 40.31846 | 9.99360 | 3 | 1.09861 | 0.27838 | 0.98852 |
| 231 | 40.31720 | 9.34744 | 0 | not available | 0.08935 | 0.98764 |
| 232 | 40.16896 | 14.89880 | 0 | not available | 0.20278 | 0.99697 |
| 233 | 40.15678 | 15.13236 | 0 | not available | 0.36211 | 0.98709 |
| 234** | 40.14413 | 15.36578 | 0 | not available | 0.78604 | 0.98641 |
| 235 | 40.13102 | 15.59905 | 0 | not available | 0.53222 | 0.98556 |
| 236 | 40.11744 | 15.83217 | 0 | not available | 0.31923 | 0.98763 |
| 237*** | 40.10339 | 16.06512 | 257 | 2.52507 | 0.75342 | 0.98992 |
| 238*** | 40.08888 | 16.29791 | 266 | 2.3731 | 0.87102 | 0.99097 |
| 239 | 40.07391 | 16.53052 | 0 | not available | 0.41237 | 0.98866 |
| 240 | 40.05848 | 16.76296 | 0 | not available | 0.00479 | 0.99887 |
| 241 | 39.97442 | 17.92232 | 0 | not available | 0.13879 | 0.98597 |
| 242 | 39.95624 | 18.15359 | 0 | not available | 0.07767 | 0.91483 |
| 243 | 39.93760 | 18.38465 | 0 | not available | 0.03602 | 0.94887 |
| 244 | 40.49609 | 8.84406 | 0 | not available | 0.03577 | 0.99932 |
| 245** | 40.49772 | 8.20406 | 3 | 1.09861 | 0.16509 | 0.98342 |
| 246 | 40.49887 | 8.56422 | 0 | not available | 0.25431 | 0.99426 |
| 247** | 40.49953 | 8.92449 | 6 | 1.56071 | 0.05681 | 0.98433 |
| 248** | 40.49972 | 9.28578 | 3 | 0.63651 | 0.16366 | 0.98717 |
| 249 | 40.49942 | 9.64669 | 3 | 0.63651 | not available | 0.98718 |
| 250 | 40.49864 | 9.00694 | 0 | not available | 0.16468 | 0.9958 |
| 251 | 40.49737 | 9.36706 | 0 | not available | 0.12155 | 0.98145 |
| 252 | 40.34818 | 14.91444 | 0 | not available | 0.24941 | 0.97417 |
| 253 | 40.33593 | 15.14862 | 0 | not available | 0.5719 | 0.98738 |
| 254** | 40.32320 | 15.38264 | 0 | not available | 0.99294 | 0.99568 |
| 255 | 40.31001 | 15.61652 | 0 | not available | 0.64952 | 0.97994 |
| 256 | 40.29634 | 15.85024 | 0 | not available | 0.40204 | 0.97935 |
| 257*** | 40.28221 | 16.08380 | 35 | 2.21364 | 0.47047 | 0.99546 |
| 258 | 40.26761 | 16.31719 | 0 | not available | 0.0683 | 0.99421 |
| 259 | 40.25254 | 16.55040 | 0 | not available | 0.00745 | 0.98924 |
| 260 | 40.23701 | 16.78344 | 0 | not available | 0.06056 | 0.98236 |
| 261 | 40.15242 | 17.94577 | 0 | not available | 0.23 | 0.97458 |
| 262 | 40.13413 | 18.17762 | 0 | not available | 0.00007 | 0.91624 |
| 263 | 40.11537 | 18.40926 | 0 | not available | 0.07874 | 0.94577 |
| 264 | 40.67625 | 8.81946 | 0 | not available | 0.10353 | 0.99782 |
| 265 | 40.67789 | 8.18579 | 0 | not available | 0.0915 | 0.98223 |
| 266 | 40.67904 | 8.55229 | 1 | not available | not available | 0.9255 |
| 267 | 40.67971 | 8.91890 | 0 | not available | 0.11994 | 0.99297 |
| 268 | 40.67989 | 9.28655 | 0 | not available | 0.46534 | 0.98685 |
| 269 | 40.67959 | 9.65381 | 0 | not available | 0.05012 | 0.99578 |
| 270 | 40.67881 | 9.02040 | 0 | not available | not available | 0.9977 |
| 271 | 40.67754 | 9.38686 | 0 | not available | 0.00037 | 0.97902 |
| 272 | 40.56155 | 14.22502 | 0 | not available | 0.12252 | 0.99664 |
| 273 | 40.55065 | 14.46022 | 0 | not available | 0.16629 | 0.98057 |
| 274 | 40.53926 | 14.69529 | 0 | not available | 0.00217 | 0.9988 |
| 275 | 40.52740 | 14.93022 | 0 | not available | 0.03514 | 0.95406 |
| 276 | 40.51507 | 15.16501 | 0 | not available | 0.32294 | 0.98572 |
| 277** | 40.50226 | 15.39966 | 0 | not available | 0.67286 | 0.98674 |
| 278 | 40.48898 | 15.63415 | 0 | not available | 0.33088 | 0.9799 |
| 279 | 40.47523 | 15.86848 | 0 | not available | 0.35001 | 0.99673 |
| 280 | 40.46101 | 16.10264 | 0 | not available | 0.59214 | 0.99529 |
| 281 | 40.44632 | 16.33664 | 0 | not available | 0.00153 | 0.9943 |
| 282 | 40.43116 | 16.57046 | 0 | not available | 0.01394 | 0.98182 |
| 283 | 40.41553 | 16.80410 | 0 | not available | 0.05502 | 0.97837 |
| 284 | 40.39943 | 17.03756 | 0 | not available | 0.03819 | 0.99933 |
| 285 | 40.38287 | 17.27082 | 0 | not available | 0.04647 | 0.90434 |
| 286 | 40.36585 | 17.50389 | 0 | not available | 0.06712 | 0.94265 |
| 287 | 40.34836 | 17.73676 | 0 | not available | 0.22262 | 0.93269 |
| 288 | 40.33041 | 17.96942 | 0 | not available | 0.03689 | 0.91309 |
| 289 | 40.31200 | 18.20187 | 0 | not available | 0.02419 | 0.87061 |
| 290 | 40.29313 | 18.43410 | 0 | not available | 0.10632 | 0.98574 |
| 291 | 40.85640 | 8.79463 | 0 | not available | 0.02795 | 0.99945 |
| 292 | 40.85805 | 8.16736 | 0 | not available | 0.53747 | 0.94727 |
| 293 | 40.85921 | 8.54026 | 0 | not available | 0.61579 | 0.98143 |
| 294** | 40.85988 | 8.91327 | 12 | 2.02281 | 0.12064 | 0.97748 |
| 295 | 40.86007 | 9.28733 | 0 | not available | 0.14667 | 0.98883 |
| 296 | 40.85976 | 9.66099 | 0 | not available | 0.25446 | 0.98539 |
| 297 | 40.85897 | 9.03398 | 0 | not available | 0.04195 | 0.93881 |
| 298 | 40.85769 | 9.40684 | 0 | not available | 0.45122 | 0.9792 |
| 299 | 40.78865 | 13.05814 | 0 | not available | 0.03884 | 0.9999 |
| 300 | 40.78008 | 13.29455 | 0 | not available | 0.02083 | 1 |
| 301 | 40.77102 | 13.53085 | 3 | 0 | 0.05813 | 0.99929 |
| 302 | 40.76149 | 13.76704 | 0 | not available | 0.15229 | 0.99439 |
| 303 | 40.75147 | 14.00312 | 0 | not available | 0.22393 | 0.94612 |
| 304 | 40.74098 | 14.23907 | 0 | not available | 0.01377 | 0.94389 |
| 305 | 40.73000 | 14.47490 | 0 | not available | 0.32994 | 0.83493 |
| 306 | 40.71854 | 14.71059 | 0 | not available | 0.29051 | 0.85631 |
| 307 | 40.70661 | 14.94615 | 0 | not available | 0.52971 | 0.94165 |
| 308 | 40.69420 | 15.18156 | 0 | not available | 0.5158 | 0.97947 |
| 309 | 40.68131 | 15.41682 | 0 | not available | 0.23078 | 0.98464 |
| 310 | 40.66795 | 15.65193 | 0 | not available | 0.03756 | 0.97739 |
| 311 | 40.65411 | 15.88687 | 0 | not available | 0.03625 | 0.97194 |
| 312 | 40.63980 | 16.12166 | 0 | not available | 0.19587 | 0.99255 |
| 313 | 40.62501 | 16.35626 | 0 | not available | 0.04857 | 0.99548 |
| 314 | 40.60976 | 16.59070 | 0 | not available | 0.26294 | 0.95896 |
| 315 | 40.59403 | 16.82495 | 0 | not available | 0.35987 | 0.97946 |
| 316 | 40.57784 | 17.05901 | 0 | not available | 0.33527 | 0.95877 |
| 317 | 40.56117 | 17.29288 | 0 | not available | 0.36257 | 0.89118 |
| 318 | 40.54404 | 17.52655 | 0 | not available | 0.05865 | 0.96459 |
| 319 | 40.52644 | 17.76002 | 0 | not available | 0.01178 | 0.96559 |
| 320 | 40.50838 | 17.99328 | 0 | not available | 0.13815 | 0.9606 |
| 321 | 40.48985 | 18.22633 | 0 | not available | 0.30284 | 0.98709 |
| 322 | 41.03654 | 8.76957 | 0 | not available | 0.32546 | 0.99998 |
| 323 | 41.03820 | 8.14876 | 0 | not available | 0.99127 | 0.9946 |
| 324 | 41.04005 | 8.90758 | 0 | not available | 0.95553 | 0.99596 |
| 325 | 41.04023 | 9.28811 | 0 | not available | 0.20847 | 0.98568 |
| 326 | 41.03993 | 9.66824 | 0 | not available | not available | 0.98789 |
| 327 | 41.03913 | 9.04769 | 0 | not available | 0.11209 | 0.9431 |
| 328 | 41.03784 | 9.42701 | 0 | not available | 0.07251 | 0.99922 |
| 329 | 40.97650 | 12.83201 | 0 | not available | 0.15283 | 0.99958 |
| 330 | 40.96836 | 13.06915 | 0 | not available | 0.17213 | 0.99638 |
| 331 | 40.93095 | 14.01667 | 0 | not available | 0.05979 | 0.89596 |
| 332 | 40.92039 | 14.25325 | 0 | not available | 0.07159 | 0.49253 |
| 333 | 40.90934 | 14.48971 | 0 | not available | 0.20828 | 0.77595 |
| 334 | 40.89782 | 14.72603 | 5 | 0 | 0.46027 | 0.90722 |
| 335** | 40.88581 | 14.96222 | 29 | 0 | 0.45872 | 0.96038 |
| 336 | 40.87332 | 15.19825 | 4 | 0 | 0.37465 | 0.97983 |
| 337 | 40.86035 | 15.43414 | 7 | 0 | 0.17173 | 0.98414 |
| 338 | 40.84690 | 15.66987 | 0 | not available | 0.02651 | 0.98713 |
| 339 | 40.83298 | 15.90544 | 0 | not available | 0.00197 | 0.99193 |
| 340 | 40.81858 | 16.14084 | 0 | not available | not available | 0.99254 |
| 341 | 40.80370 | 16.37607 | 0 | not available | 0.28918 | 0.98054 |
| 342 | 40.78835 | 16.61112 | 0 | not available | 0.52102 | 0.96742 |
| 343 | 40.77252 | 16.84598 | 0 | not available | 0.54076 | 0.96839 |
| 344 | 40.75622 | 17.08066 | 0 | not available | 0.57939 | 0.98978 |
| 345 | 40.73945 | 17.31514 | 0 | not available | 0.4079 | 0.97689 |
| 346 | 40.72221 | 17.54942 | 0 | not available | 0.08131 | 0.96221 |
| 347 | 40.70450 | 17.78350 | 0 | not available | 0.16935 | 0.98052 |
| 348 | 40.68633 | 18.01736 | 0 | not available | 0.12524 | 0.93393 |
| 349 | 41.22039 | 9.28890 | 0 | not available | 0.85337 | 0.99963 |
| 350 | 41.22008 | 9.67555 | 0 | not available | 0.51216 | 0.96915 |
| 351 | 41.21928 | 9.06152 | 0 | not available | 0.811 | 0.96438 |
| 352 | 41.14807 | 13.08026 | 0 | not available | 0.05204 | 0.99623 |
| 353 | 41.13022 | 13.55553 | 0 | not available | 0.00329 | 0.99383 |
| 354 | 41.12057 | 13.79299 | 0 | not available | 0.0473 | 0.97594 |
| 355 | 41.11042 | 14.03034 | 0 | not available | 0.07509 | 0.94888 |
| 356 | 41.09979 | 14.26756 | 0 | not available | 0.07582 | 0.82266 |
| 357 | 41.08868 | 14.50466 | 0 | not available | 0.34667 | 0.93917 |
| 358 | 41.07708 | 14.74161 | 3 | 0 | 0.13002 | 0.92345 |
| 359 | 41.06499 | 14.97843 | 10 | 0 | not available | 0.9693 |
| 360 | 41.05243 | 15.21510 | 0 | not available | 0.09953 | 0.97837 |
| 361 | 41.03938 | 15.45162 | 0 | not available | 0.14937 | 0.99066 |
| 362 | 41.02584 | 15.68798 | 0 | not available | 0.13379 | 0.96416 |
| 363 | 41.01183 | 15.92417 | 0 | not available | 0.05061 | 0.98834 |
| 364 | 40.99734 | 16.16020 | 0 | not available | 0.52629 | 0.9825 |
| 365** | 40.98237 | 16.39605 | 0 | not available | 0.84892 | 0.9966 |
| 366 | 40.96692 | 16.63172 | 0 | not available | 0.34086 | 0.97543 |
| 367 | 40.95099 | 16.86721 | 0 | not available | 0.02623 | 0.95053 |
| 368 | 40.93459 | 17.10250 | 0 | not available | 0.01379 | 0.96249 |
| 369 | 40.91772 | 17.33760 | 0 | not available | 0.20532 | 0.97165 |
| 370 | 40.90037 | 17.57249 | 0 | not available | 0.15039 | 0.99931 |
| 371 | 41.40024 | 9.68293 | 0 | not available | 0.11941 | 0.72611 |
| 372 | 41.34376 | 12.61451 | 0 | not available | 0.00015 | 1 |
| 373 | 41.33601 | 12.85304 | 0 | not available | 0.29018 | 0.98899 |
| 374 | 41.32777 | 13.09147 | 0 | not available | 0.34923 | 0.94798 |
| 375** | 41.31903 | 13.32981 | 30 | 0.71551 | 0.44873 | 0.96924 |
| 376*** | 41.30981 | 13.56803 | 7 | 0.9557 | 0.64283 | 0.96911 |
| 377 | 41.30009 | 13.80614 | 1 | not available | 0.10215 | 0.96318 |
| 378 | 41.28988 | 14.04414 | 0 | not available | 0.25298 | 0.97041 |
| 379** | 41.27919 | 14.28200 | 80 | 1.69313 | 0.18546 | 0.98182 |
| 380*** | 41.26800 | 14.51974 | 35 | 1.02201 | 0.40681 | 0.97493 |
| 381 | 41.25633 | 14.75734 | 0 | not available | 0.11511 | 0.97624 |
| 382 | 41.24417 | 14.99479 | 1 | not available | 0.03534 | 0.98594 |
| 383 | 41.23152 | 15.23210 | 1 | not available | 0.15455 | 0.98988 |
| 384 | 41.21839 | 15.46925 | 0 | not available | 0.04914 | 0.99418 |
| 385 | 41.20477 | 15.70625 | 0 | not available | 0.10546 | 0.99582 |
| 386 | 41.19067 | 15.94308 | 0 | not available | 0.19613 | 0.97055 |
| 387 | 41.17609 | 16.17973 | 0 | not available | 0.32254 | 0.96836 |
| 388 | 41.16102 | 16.41621 | 0 | not available | 0.01774 | 0.94439 |
| 389 | 41.14548 | 16.65251 | 0 | not available | 0.0401 | 0.91304 |
| 390 | 41.12945 | 16.88862 | 0 | not available | 0.09358 | 0.81417 |
| 391 | 41.11295 | 17.12454 | 0 | not available | 0.0866 | 0.9886 |
| 392 | 41.53085 | 12.38523 | 0 | not available | 0.03467 | 0.99445 |
| 393 | 41.52355 | 12.62451 | 7 | 0 | 0.03292 | 0.8473 |
| 394 | 41.51575 | 12.86370 | 19 | 0.85394 | 0.02579 | 0.90934 |
| 395** | 41.50746 | 13.10279 | 2 | 0.69315 | 0.4806 | 0.97748 |
| 396 | 41.49867 | 13.34177 | 1 | not available | 0.4903 | 0.98625 |
| 397** | 41.48938 | 13.58065 | 20 | 1.00976 | 0.19417 | 0.98259 |
| 398** | 41.47961 | 13.81941 | 10 | 1.0889 | 0.0455 | 0.95261 |
| 399*** | 41.46934 | 14.05806 | 22 | 2.51558 | 0.25687 | 0.9795 |
| 400*** | 41.45857 | 14.29657 | 71 | 1.56684 | 0.73803 | 0.99317 |
| 401*** | 41.44732 | 14.53496 | 6 | 1.79176 | 0.53296 | 0.98273 |
| 402** | 41.43557 | 14.77320 | 18 | 1.43575 | 0.10075 | 0.99194 |
| 403 | 41.42333 | 15.01130 | 0 | not available | 0.1268 | 0.99302 |
| 404 | 41.41061 | 15.24925 | 0 | not available | 0.12224 | 0.99312 |
| 405 | 41.39739 | 15.48705 | 0 | not available | 0.04148 | 0.94635 |
| 406 | 41.38369 | 15.72468 | 0 | not available | 0.02509 | 0.97384 |
| 407 | 41.36950 | 15.96215 | 0 | not available | 0.19131 | 0.98596 |
| 408 | 41.35483 | 16.19944 | 0 | not available | 0.19193 | 0.96154 |
| 409 | 41.33967 | 16.43656 | 0 | not available | 0.03707 | 0.97001 |
| 410 | 41.71752 | 12.15463 | 0 | not available | 0.02861 | 0.94889 |
| 411 | 41.71068 | 12.39466 | 1 | not available | 0.40004 | 0.81428 |
| 412 | 41.70333 | 12.63460 | 1 | not available | 0.16134 | 0.88874 |
| 413 | 41.69548 | 12.87445 | 0 | not available | 0.30869 | 0.96753 |
| 414 | 41.68714 | 13.11420 | 1 | not available | 0.34026 | 0.95907 |
| 415** | 41.67829 | 13.35385 | 14 | 1.25276 | 0.13973 | 0.90867 |
| 416** | 41.66895 | 13.59339 | 32 | 1.57468 | 0.14116 | 0.95612 |
| 417*** | 41.65911 | 13.83281 | 126 | 0.98479 | 0.31235 | 0.99253 |
| 418*** | 41.64878 | 14.07211 | 6 | 1.56071 | 0.33522 | 0.99048 |
| 419*** | 41.63795 | 14.31128 | 9 | 1.58109 | 0.33443 | 0.97917 |
| 420** | 41.62662 | 14.55031 | 3 | 1.09861 | 0.114 | 0.98534 |
| 421** | 41.61480 | 14.78921 | 21 | 1.78973 | 0.1539 | 0.98406 |
| 422 | 41.60249 | 15.02796 | 1 | not available | 0.42926 | 0.99262 |
| 423 | 41.58968 | 15.26656 | 0 | not available | not available | 0.983 |
| 424 | 41.57638 | 15.50500 | 0 | not available | 0.0112 | 0.9971 |
| 425 | 41.56259 | 15.74328 | 0 | not available | 0.42348 | 0.96832 |
| 426 | 41.54832 | 15.98139 | 0 | not available | 0.13686 | 0.97482 |
| 427 | 41.90378 | 11.92270 | 0 | not available | 0.05195 | 0.99777 |
| 428 | 41.89739 | 12.16348 | 0 | not available | 0.19947 | 0.93468 |
| 429** | 41.89050 | 12.40417 | 49 | 2.48773 | 0.19379 | 0.53563 |
| 430 | 41.88311 | 12.64479 | 2 | 0 | 0.12604 | 0.61667 |
| 431 | 41.87521 | 12.88530 | 2 | 0 | 0.05404 | 0.96528 |
| 432*** | 41.86681 | 13.12572 | 7 | 1.35178 | 0.34857 | 0.96603 |
| 433** | 41.85791 | 13.36603 | 0 | not available | 0.68358 | 0.99213 |
| 434** | 41.84851 | 13.60624 | 1 | not available | 0.66436 | 0.99654 |
| 435** | 41.83861 | 13.84632 | 2 | 0.69315 | 0.89021 | 0.99693 |
| 436 | 41.82821 | 14.08628 | 0 | not available | 0.38427 | 0.98611 |
| 437*** | 41.81731 | 14.32611 | 35 | 2.02728 | 0.29942 | 0.99421 |
| 438** | 41.80591 | 14.56581 | 11 | 1.76776 | 0.18015 | 0.9961 |
| 439*** | 41.79402 | 14.80537 | 25 | 2.01253 | 0.61423 | 0.99604 |
| 440** | 41.78163 | 15.04477 | 6 | 0.86756 | 0.083 | 0.99065 |
| 441 | 41.76874 | 15.28403 | 0 | not available | 0.07548 | 0.96278 |
| 442 | 41.75536 | 15.52312 | 0 | not available | 0.42001 | 0.9772 |
| 443** | 41.74149 | 15.76206 | 0 | not available | 0.87105 | 0.98036 |
| 444** | 41.72712 | 16.00082 | 0 | not available | 0.70919 | 0.98764 |
| 445 | 41.71226 | 16.23940 | 0 | not available | 0.07993 | 0.99891 |
| 446 | 42.08959 | 11.68942 | 0 | not available | 0.01862 | 0.97194 |
| 447** | 42.08367 | 11.93095 | 0 | not available | 0.74489 | 0.97556 |
| 448*** | 42.07725 | 12.17240 | 6 | 1.0114 | 0.66471 | 0.97245 |
| 449 | 42.07031 | 12.41378 | 0 | not available | 0.44262 | 0.9031 |
| 450** | 42.06287 | 12.65506 | 47 | 1.09202 | 0.18333 | 0.92026 |
| 451*** | 42.05493 | 12.89625 | 23 | 1.28016 | 0.43317 | 0.98403 |
| 452 | 42.04648 | 13.13735 | 0 | not available | 0.45799 | 0.98618 |
| 453** | 42.03752 | 13.37833 | 4 | 0.56234 | 0.26411 | 0.94482 |
| 454*** | 42.02806 | 13.61920 | 14 | 1.25276 | 0.28821 | 0.97448 |
| 455*** | 42.01810 | 13.85996 | 18 | 1.62904 | 0.35307 | 0.97562 |
| 456** | 42.00763 | 14.10059 | 1 | not available | 0.77634 | 0.99317 |
| 457** | 41.99667 | 14.34109 | 7 | 1.27703 | 0.17778 | 0.9929 |
| 458** | 41.98520 | 14.58145 | 6 | 1.56071 | 0.13077 | 0.9931 |
| 459** | 41.97323 | 14.82167 | 3 | 1.09861 | 0.05616 | 0.97215 |
| 460 | 41.96076 | 15.06174 | 1 | not available | 0.06557 | 0.96413 |
| 461 | 41.94779 | 15.30165 | 0 | not available | 0.13353 | 0.99643 |
| 462 | 41.93433 | 15.54141 | 0 | not available | 0.19305 | 0.99195 |
| 463 | 41.92036 | 15.78100 | 0 | not available | 0.30405 | 0.99088 |
| 464 | 41.90591 | 16.02041 | 0 | not available | 0.57361 | 0.98927 |
| 465 | 41.89096 | 16.25965 | 0 | not available | 0.07108 | 0.99509 |
| 466 | 42.29688 | 9.99941 | 0 | not available | 0.99976 | 0.57636 |
| 467 | 42.29451 | 10.24251 | 0 | not available | 1 | 0.99998 |
| 468 | 42.28432 | 10.97002 | 0 | not available | 0.99859 | 0.99821 |
| 469 | 42.27990 | 11.21242 | 0 | not available | 0.75039 | 0.99994 |
| 470 | 42.27496 | 11.45477 | 0 | not available | 0.12059 | 0.99413 |
| 471 | 42.26952 | 11.69706 | 0 | not available | 0.12966 | 0.97882 |
| 472** | 42.26356 | 11.93927 | 7 | 0 | 0.57022 | 0.99449 |
| 473 | 42.25710 | 12.18141 | 1 | not available | 0.26611 | 0.97156 |
| 474 | 42.25012 | 12.42347 | 3 | 0.63651 | 0.04413 | 0.96653 |
| 475** | 42.24263 | 12.66543 | 5 | 0.67301 | 0.11241 | 0.98165 |
| 476** | 42.23464 | 12.90731 | 18 | 1.16409 | 0.11656 | 0.99141 |
| 477** | 42.22614 | 13.14908 | 30 | 0.63651 | 0.04212 | 0.99406 |
| 478** | 42.21712 | 13.39074 | 1 | not available | 0.67499 | 0.99149 |
| 479*** | 42.20760 | 13.63229 | 44 | 2.17033 | 0.67686 | 0.99323 |
| 480** | 42.19758 | 13.87372 | 11 | 0.88557 | 0.42698 | 0.98386 |
| 481*** | 42.18705 | 14.11502 | 6 | 1.24245 | 0.56909 | 0.9836 |
| 482 | 42.17601 | 14.35620 | 3 | 0.63651 | 0.07204 | 0.95215 |
| 483 | 42.16447 | 14.59723 | 2 | 0 | 0.03976 | 0.97067 |
| 484 | 42.15242 | 14.83812 | 0 | not available | 0.00329 | 0.99241 |
| 485 | 42.11328 | 15.55986 | 0 | not available | 0.04039 | 0.99899 |
| 486 | 42.46433 | 10.97565 | 0 | not available | 0.99988 | 0.9976 |
| 487** | 42.45988 | 11.21875 | 3 | 0.63651 | 0.61379 | 0.96756 |
| 488 | 42.45492 | 11.46179 | 0 | not available | 0.24701 | 0.99378 |
| 489** | 42.44944 | 11.70477 | 41 | 1.02531 | 0.04596 | 0.9952 |
| 490** | 42.44345 | 11.94767 | 17 | 1.00621 | 0.07047 | 0.98855 |
| 491** | 42.43694 | 12.19050 | 95 | 1.75156 | 0.0666 | 0.95593 |
| 492 | 42.42992 | 12.43325 | 0 | not available | 0.02704 | 0.97781 |
| 493** | 42.42239 | 12.67590 | 12 | 1.35221 | 0.19607 | 0.99479 |
| 494*** | 42.41434 | 12.91846 | 7 | 1.15374 | 0.3315 | 0.95997 |
| 495** | 42.40578 | 13.16092 | 2 | 0.69315 | 0.22355 | 0.99361 |
| 496*** | 42.39672 | 13.40326 | 4 | 1.03972 | 0.37309 | 0.94428 |
| 497*** | 42.38714 | 13.64550 | 10 | 1.41848 | 0.81354 | 0.99447 |
| 498** | 42.37705 | 13.88761 | 4 | 0.69315 | 0.26581 | 0.99106 |
| 499 | 42.36645 | 14.12959 | 8 | 0.56234 | 0.0077 | 0.90612 |
| 500 | 42.35534 | 14.37144 | 1 | not available | 0.01749 | 0.95646 |
| 501 | 42.34373 | 14.61315 | 0 | not available | 0.00122 | 0.9969 |
| 502 | 42.27809 | 15.81940 | 0 | not available | 0.00482 | 0.22296 |
| 503 | 42.65706 | 10.00569 | 0 | not available | 0.99999 | 0.99925 |
| 504 | 42.65466 | 10.24966 | 0 | not available | 0.99414 | 0.99935 |
| 505 | 42.65174 | 10.49359 | 0 | not available | 0.99248 | 0.99516 |
| 506** | 42.64434 | 10.98134 | 0 | not available | 0.89563 | 0.99168 |
| 507** | 42.63986 | 11.22514 | 4 | 1.03972 | 0.1121 | 0.99184 |
| 508 | 42.63487 | 11.46888 | 0 | not available | 0.07981 | 0.99726 |
| 509*** | 42.62936 | 11.71255 | 28 | 1.49688 | 0.2224 | 0.99286 |
| 510*** | 42.62332 | 11.95615 | 315 | 1.47417 | 0.34251 | 0.98346 |
| 511** | 42.61678 | 12.19968 | 178 | 0.69905 | 0.12222 | 0.99029 |
| 512** | 42.60971 | 12.44312 | 66 | 1.85565 | 0.19388 | 0.96697 |
| 513*** | 42.60213 | 12.68647 | 3 | 1.09861 | 0.20875 | 0.92673 |
| 514*** | 42.59404 | 12.92972 | 14 | 0.89533 | 0.43624 | 0.99757 |
| 515** | 42.58542 | 13.17286 | 12 | 1.02818 | 0.09328 | 0.99438 |
| 516** | 42.57630 | 13.41590 | 2 | 0.69315 | 0.97612 | 0.99854 |
| 517* | 42.56666 | 13.65882 | 7 | 1.00424 | 0.22521 | 0.98951 |
| 518 | 42.55651 | 13.90162 | 2 | 0.69315 | 0.02762 | 0.98784 |
| 519 | 42.54584 | 14.14429 | 1 | not available | 0.08882 | 0.91586 |
| 520 | 42.83714 | 10.00861 | 0 | not available | 0.99848 | 0.9999 |
| 521** | 42.83473 | 10.25328 | 3 | 0 | 0.85107 | 0.96862 |
| 522 | 42.83179 | 10.49792 | 0 | not available | 0.95056 | 0.98492 |
| 523** | 42.82833 | 10.74253 | 0 | not available | 0.7926 | 0.98654 |
| 524 | 42.82434 | 10.98708 | 0 | not available | 0.13776 | 0.96741 |
| 525** | 42.81984 | 11.23158 | 3 | 1.09861 | 0.08443 | 0.97185 |
| 526*** | 42.81481 | 11.47603 | 4 | 1.03972 | 0.24782 | 0.98894 |
| 527** | 42.80926 | 11.72041 | 15 | 0.62771 | 0.15357 | 0.98553 |
| 528** | 42.80319 | 11.96471 | 9 | 1.31078 | 0.18181 | 0.9864 |
| 529** | 42.79661 | 12.20894 | 3 | 0.63651 | 0.19582 | 0.98874 |
| 530** | 42.78950 | 12.45308 | 35 | 1.98834 | 0.06512 | 0.97862 |
| 531 | 42.78187 | 12.69713 | 9 | 0.68696 | 0.06857 | 0.96195 |
| 532*** | 42.77372 | 12.94108 | 14 | 1.90854 | 0.20904 | 0.99808 |
| 533*** | 42.76506 | 13.18492 | 45 | 1.95907 | 0.64873 | 0.99314 |
| 534** | 42.75587 | 13.42866 | 1 | not available | 0.61183 | 0.99383 |
| 535** | 42.74617 | 13.67227 | 5 | 0.67301 | 0.1979 | 0.96768 |
| 536 | 42.73596 | 13.91577 | 1 | not available | 0.02801 | 0.93738 |
| 537 | 42.72523 | 14.15913 | 0 | not available | not available | 0.99588 |
| 538 | 43.01913 | 9.66137 | 0 | not available | 0.99917 | 0.99894 |
| 539 | 43.01183 | 10.50229 | 0 | not available | 0.64152 | 0.94503 |
| 540 | 43.00835 | 10.74761 | 0 | not available | 0.2473 | 0.97125 |
| 541** | 43.00434 | 10.99288 | 3 | 1.09861 | 0.06374 | 0.9844 |
| 542** | 42.99981 | 11.23809 | 2 | 0.69315 | 0.20517 | 0.98872 |
| 543 | 42.99475 | 11.48324 | 0 | not available | 0.0405 | 0.99701 |
| 544*** | 42.98916 | 11.72833 | 3 | 1.09861 | 0.28296 | 0.98904 |
| 545** | 42.98306 | 11.97335 | 3 | 1.09861 | 0.12993 | 0.97028 |
| 546** | 42.97643 | 12.21828 | 4 | 1.38629 | 0.04275 | 0.9775 |
| 547** | 42.96927 | 12.46313 | 67 | 2.11169 | 0.06514 | 0.95163 |
| 548** | 42.96160 | 12.70788 | 50 | 1.89681 | 0.09508 | 0.93196 |
| 549 | 42.95340 | 12.95254 | 0 | not available | 0.32185 | 0.99621 |
| 550** | 42.94468 | 13.19709 | 1 | not available | 0.88108 | 0.99618 |
| 551 | 42.93544 | 13.44153 | 0 | not available | 0.1212 | 0.99355 |
| 552** | 42.92568 | 13.68585 | 11 | 1.36671 | 0.03015 | 0.95143 |
| 553 | 42.91540 | 13.93004 | 1 | not available | 0.00786 | 0.9332 |
| 554 | 43.19187 | 10.50670 | 0 | not available | 0.64495 | 0.98019 |
| 555** | 43.18836 | 10.75274 | 2 | 0.69315 | 0.22606 | 0.99738 |
| 556 | 43.18433 | 10.99872 | 0 | not available | 0.08232 | 0.99109 |
| 557 | 43.17977 | 11.24466 | 0 | not available | 0.42028 | 0.9858 |
| 558 | 43.17468 | 11.49053 | 0 | not available | 0.08989 | 0.98123 |
| 559 | 43.16906 | 11.73633 | 0 | not available | not available | 0.96646 |
| 560*** | 43.16291 | 11.98206 | 23 | 2.23819 | 0.25414 | 0.97533 |
| 561*** | 43.15624 | 12.22771 | 14 | 1.16308 | 0.29006 | 0.93658 |
| 562** | 43.14904 | 12.47327 | 26 | 2.00793 | 0.0732 | 0.88383 |
| 563** | 43.14132 | 12.71874 | 14 | 2.22056 | 0.18545 | 0.98024 |
| 564 | 43.13307 | 12.96411 | 3 | 0 | 0.19442 | 0.98597 |
| 565** | 43.12430 | 13.20937 | 5 | 0.67301 | 0.30216 | 0.98606 |
| 566 | 43.11500 | 13.45452 | 3 | 0 | 0.00793 | 0.97592 |
| 567 | 43.10518 | 13.69955 | 6 | 1.24245 | 0.00366 | 0.95288 |
| 568 | 43.09483 | 13.94445 | 2 | 0 | 0.00439 | 0.99051 |
| 569 | 43.37928 | 9.70657 | 0 | not available | 0.89949 | 1 |
| 570 | 43.37735 | 10.01752 | 0 | not available | 1 | 0.99999 |
| 571 | 43.37489 | 10.26435 | 0 | not available | 0.99041 | 0.99731 |
| 572*** | 43.37190 | 10.51115 | 4 | 1.03972 | 0.24207 | 0.95474 |
| 573 | 43.36837 | 10.75791 | 2 | 0 | 0.03952 | 0.99048 |
| 574 | 43.36431 | 11.00463 | 0 | not available | 0.10224 | 0.99239 |
| 575** | 43.35972 | 11.25128 | 6 | 0.69315 | 0.30552 | 0.95799 |
| 576 | 43.35460 | 11.49788 | 1 | not available | 0.07624 | 0.98919 |
| 577 | 43.34895 | 11.74441 | 0 | not available | 0.0004 | 0.96817 |
| 578 | 43.34276 | 11.99086 | 3 | 0 | 0.11234 | 0.97987 |
| 579** | 43.33605 | 12.23723 | 16 | 2.18732 | 0.02089 | 0.98352 |
| 580** | 43.32880 | 12.48351 | 14 | 1.33166 | 0.05133 | 0.98153 |
| 581*** | 43.32103 | 12.72970 | 12 | 1.63263 | 0.29023 | 0.97198 |
| 582*** | 43.31273 | 12.97579 | 6 | 1.32966 | 0.294 | 0.96528 |
| 583** | 43.30390 | 13.22177 | 5 | 1.33218 | 0.01983 | 0.97473 |
| 584** | 43.29455 | 13.46763 | 9 | 1.30309 | 0.04499 | 0.94326 |
| 585 | 43.28466 | 13.71337 | 2 | 0 | not available | 0.92206 |
| 586 | 43.55493 | 10.26811 | 0 | not available | 0.71941 | 0.90034 |
| 587 | 43.55192 | 10.51565 | 12 | 0.83699 | 0.01578 | 0.96371 |
| 588 | 43.54837 | 10.76314 | 8 | 1.07354 | not available | 0.98752 |
| 589 | 43.54429 | 11.01058 | 4 | 1.03972 | 0.01278 | 0.97237 |
| 590** | 43.53967 | 11.25797 | 7 | 1.54983 | 0.03043 | 0.97329 |
| 591** | 43.53451 | 11.50530 | 8 | 1.55958 | 0.1421 | 0.93376 |
| 592 | 43.52883 | 11.75256 | 0 | not available | 0.10083 | 0.95296 |
| 593 | 43.52260 | 11.99974 | 0 | not available | 0.05839 | 0.9683 |
| 594** | 43.51585 | 12.24684 | 43 | 1.64853 | 0.14756 | 0.96404 |
| 595** | 43.50856 | 12.49385 | 2 | 0.69315 | 0.3911 | 0.99281 |
| 596 | 43.50074 | 12.74076 | 1 | not available | 0.33272 | 0.9791 |
| 597** | 43.49238 | 12.98757 | 3 | 1.09861 | 0.16111 | 0.97732 |
| 598 | 43.48350 | 13.23428 | 4 | 1.03972 | 0.00844 | 0.94206 |
| 599 | 43.47409 | 13.48086 | 0 | not available | 0.07621 | 0.92331 |
| 600 | 43.46414 | 13.72732 | 0 | not available | 0.03599 | 0.97338 |
| 601 | 43.73272 | 7.39892 | 0 | not available | 0.4372 | 0.40361 |
| 602 | 43.73562 | 7.88176 | 0 | not available | 0.9354 | 0.98138 |
| 603** | 43.73497 | 10.27191 | 0 | not available | 0.77923 | 0.95083 |
| 604 | 43.73194 | 10.52018 | 0 | not available | 0.24656 | 0.86723 |
| 605** | 43.72837 | 10.76841 | 6 | 0.86756 | 0.20753 | 0.90338 |
| 606 | 43.72426 | 11.01660 | 4 | 1.38629 | 0.01552 | 0.9083 |
| 607** | 43.71961 | 11.26472 | 10 | 1.83437 | 0.01146 | 0.82141 |
| 608 | 43.71442 | 11.51279 | 2 | 0 | 0.1353 | 0.96234 |
| 609** | 43.70870 | 11.76078 | 11 | 1.84622 | 0.14856 | 0.97199 |
| 610 | 43.70244 | 12.00870 | 0 | not available | 0.27644 | 0.99529 |
| 611 | 43.69564 | 12.25653 | 0 | not available | 0.26224 | 0.99377 |
| 612 | 43.68830 | 12.50428 | 0 | not available | 0.05778 | 0.97983 |
| 613 | 43.68043 | 12.75192 | 0 | not available | 0.17937 | 0.97274 |
| 614 | 43.67203 | 12.99947 | 3 | 0.63651 | 0.01951 | 0.96344 |
| 615 | 43.66309 | 13.24690 | 7 | 0.59827 | not available | 0.93267 |
| 616 | 43.65362 | 13.49422 | 5 | 1.05492 | 0.03752 | 0.93636 |
| 617 | 43.91274 | 7.35497 | 0 | not available | 0.13535 | 0.38351 |
| 618** | 43.91566 | 7.84527 | 2 | 0.69315 | 0.32705 | 0.9867 |
| 619** | 43.91804 | 8.33592 | 2 | 0.69315 | 0.38392 | 0.95701 |
| 620 | 43.91987 | 8.82683 | 0 | not available | 0.94378 | 0.99408 |
| 621 | 43.91751 | 10.02668 | 0 | not available | 0.97842 | 0.98329 |
| 622 | 43.91500 | 10.27574 | 0 | not available | 0.36311 | 0.84903 |
| 623 | 43.91195 | 10.52476 | 18 | 0.96089 | 0.00338 | 0.88447 |
| 624 | 43.90836 | 10.77373 | 3 | 1.09861 | 0.04736 | 0.9155 |
| 625 | 43.90422 | 11.02266 | 1 | not available | 0.10347 | 0.80508 |
| 626 | 43.89954 | 11.27154 | 1 | not available | 0.19443 | 0.9282 |
| 627 | 43.89432 | 11.52034 | 5 | 0 | 0.0443 | 0.9855 |
| 628** | 43.88856 | 11.76908 | 1 | not available | 0.70041 | 0.99723 |
| 629** | 43.88226 | 12.01774 | 4 | 0.69315 | 0.11322 | 0.99035 |
| 630 | 43.87542 | 12.26632 | 1 | not available | 0.26835 | 0.97588 |
| 631 | 43.86804 | 12.51480 | 8 | 0 | 0.1979 | 0.86365 |
| 632** | 43.86012 | 12.76319 | 5 | 1.33218 | 0.15227 | 0.93864 |
| 633 | 43.85167 | 13.01147 | 1 | not available | 0.06059 | 0.9229 |
| 634 | 43.84267 | 13.25964 | 0 | not available | not available | 0.99358 |
| 635 | 44.08927 | 7.81318 | 0 | not available | 0.15726 | 0.15612 |
| 636 | 44.09275 | 7.31060 | 0 | not available | 0.15913 | 0.21764 |
| 637 | 44.09569 | 7.80845 | 0 | not available | 0.49832 | 0.88147 |
| 638** | 44.09808 | 8.30663 | 5 | 0.5004 | 0.22534 | 0.98738 |
| 639** | 44.09993 | 8.80509 | 1 | not available | 0.75158 | 0.9531 |
| 640 | 44.10123 | 8.30375 | 0 | not available | 0.79519 | 0.98746 |
| 641** | 44.10096 | 9.30092 | 2 | 0.69315 | 0.95819 | 0.99719 |
| 642*** | 44.09953 | 9.79954 | 15 | 1.63509 | 0.64238 | 0.94321 |
| 643** | 44.09755 | 10.02979 | 3 | 1.09861 | 0.29199 | 0.81181 |
| 644*** | 44.09503 | 10.27960 | 33 | 1.84968 | 0.44275 | 0.96571 |
| 645** | 44.09196 | 10.52938 | 57 | 1.96575 | 0.18639 | 0.97132 |
| 646 | 44.08834 | 10.77911 | 0 | not available | 0.37742 | 0.98227 |
| 647 | 44.08418 | 11.02879 | 5 | 0 | 0.35877 | 0.98425 |
| 648 | 44.07947 | 11.27841 | 0 | not available | 0.09591 | 0.97102 |
| 649 | 44.07422 | 11.52797 | 0 | not available | 0.20052 | 0.99157 |
| 650 | 44.06842 | 11.77746 | 1 | not available | 0.06946 | 0.99577 |
| 651 | 44.06208 | 12.02687 | 2 | 0.69315 | 0.02371 | 0.98697 |
| 652 | 44.05520 | 12.27620 | 10 | 0.32508 | 0.0449 | 0.94702 |
| 653 | 44.04777 | 12.52543 | 5 | 0.5004 | 0.02423 | 0.85091 |
| 654 | 44.03981 | 12.77456 | 0 | not available | 0.02458 | 0.96031 |
| 655 | 44.26519 | 7.25625 | 0 | not available | 0.41001 | 0.53047 |
| 656 | 44.26925 | 7.76079 | 0 | not available | 0.53792 | 0.97211 |
| 657 | 44.27275 | 7.26582 | 2 | 0 | 0.18367 | 0.96144 |
| 658 | 44.27571 | 7.77127 | 0 | not available | 0.17305 | 0.98463 |
| 659 | 44.27812 | 8.27707 | 5 | 0 | 0.09717 | 0.99071 |
| 660 | 44.27998 | 8.78315 | 0 | not available | 0.26203 | 0.97809 |
| 661*** | 44.28128 | 8.28943 | 7 | 0.9557 | 0.72318 | 0.93671 |
| 662 | 44.28225 | 9.30351 | 0 | not available | 0.98738 | 0.99458 |
| 663** | 44.28191 | 9.81070 | 2 | 0.69315 | 0.72799 | 0.95237 |
| 664 | 44.28102 | 9.31709 | 1 | not available | 0.36512 | 0.98208 |
| 665** | 44.27958 | 9.82333 | 4 | 1.38629 | 0.09433 | 0.99089 |
| 666 | 44.27759 | 10.03293 | 7 | 0 | 0.09402 | 0.97132 |
| 667 | 44.27505 | 10.28350 | 0 | not available | 0.41878 | 0.99405 |
| 668 | 44.27196 | 10.53404 | 0 | not available | 0.26515 | 0.99561 |
| 669 | 44.26832 | 10.78453 | 0 | not available | 0.10721 | 0.98551 |
| 670 | 44.26413 | 11.03497 | 9 | 0 | 0.07373 | 0.98064 |
| 671 | 44.25939 | 11.28535 | 0 | not available | 0.24194 | 0.97854 |
| 672 | 44.25411 | 11.53567 | 0 | not available | 0.12276 | 0.99473 |
| 673** | 44.24828 | 11.78592 | 49 | 1.29726 | 0.1417 | 0.95536 |
| 674 | 44.24190 | 12.03608 | 6 | 1.10114 | 0.02803 | 0.92103 |
| 675 | 44.23497 | 12.28616 | 0 | not available | 0.03402 | 0.92703 |
| 676 | 44.22750 | 12.53615 | 0 | not available | not available | 0.98132 |
| 677 | 44.44051 | 6.68407 | 0 | not available | 0.03763 | 0.03746 |
| 678 | 44.44514 | 7.19571 | 0 | not available | 0.46382 | 0.96839 |
| 679 | 44.44923 | 7.70792 | 0 | not available | 0.00714 | 0.98999 |
| 680 | 44.45275 | 7.22062 | 3 | 0 | 0.0085 | 0.94289 |
| 681 | 44.45573 | 7.73375 | 1 | not available | 0.05632 | 0.96067 |
| 682 | 44.45815 | 8.24723 | 0 | not available | 0.01234 | 0.97898 |
| 683 | 44.46002 | 8.76100 | 2 | 0 | 0.12269 | 0.98082 |
| 684 | 44.46134 | 8.27497 | 1 | not available | 0.38804 | 0.99223 |
| 685 | 44.46210 | 8.78907 | 0 | not available | 0.60909 | 0.9095 |
| 686** | 44.46231 | 9.30444 | 14 | 1.77214 | 0.15137 | 0.88917 |
| 687 | 44.46197 | 9.81933 | 0 | not available | 0.19101 | 0.99035 |
| 688 | 44.46107 | 9.33341 | 0 | not available | 0.2375 | 0.99623 |
| 689 | 44.45962 | 9.84734 | 0 | not available | 0.07641 | 0.98491 |
| 690 | 44.45762 | 10.03610 | 0 | not available | 0.19683 | 0.99676 |
| 691 | 44.45506 | 10.28744 | 0 | not available | 0.16931 | 0.99136 |
| 692 | 44.45195 | 10.53874 | 1 | not available | 0.03192 | 0.97166 |
| 693 | 44.44829 | 10.79000 | 0 | not available | 0.02887 | 0.94751 |
| 694 | 44.44407 | 11.04121 | 1 | not available | 0.05427 | 0.94944 |
| 695 | 44.43931 | 11.29236 | 8 | 0.97431 | 0.14373 | 0.78888 |
| 696 | 44.43399 | 11.54344 | 0 | not available | 0.07054 | 0.94869 |
| 697 | 44.42812 | 11.79445 | 6 | 0 | 0.01379 | 0.92991 |
| 698 | 44.42170 | 12.04538 | 7 | 0.41012 | 0.00672 | 0.95417 |
| 699 | 44.41473 | 12.29623 | 3 | 0.63651 | 0.11909 | 0.90027 |
| 700 | 44.62043 | 6.61522 | 0 | not available | 0.00879 | 0.00926 |
| 701 | 44.62509 | 7.13460 | 0 | not available | 0.1765 | 0.71744 |
| 702 | 44.62920 | 7.65455 | 0 | not available | 0.00777 | 0.9888 |
| 703 | 44.63275 | 7.17500 | 0 | not available | 0.0034 | 0.9704 |
| 704 | 44.63574 | 7.69588 | 7 | 0.41012 | 0.0295 | 0.941 |
| 705 | 44.63818 | 8.21712 | 0 | not available | 0.00349 | 0.96364 |
| 706 | 44.64006 | 8.73864 | 0 | not available | 0.01953 | 0.98684 |
| 707 | 44.64138 | 8.26037 | 0 | not available | 0.00287 | 0.98193 |
| 708 | 44.64215 | 8.78224 | 0 | not available | 0.23046 | 0.97184 |
| 709 | 44.64236 | 9.30538 | 0 | not available | 0.22888 | 0.97585 |
| 710 | 44.64202 | 9.82805 | 0 | not available | 0.34393 | 0.99937 |
| 711 | 44.64111 | 9.34989 | 0 | not available | 0.07717 | 0.99796 |
| 712 | 44.63965 | 9.87158 | 0 | not available | 0.14521 | 0.99662 |
| 713 | 44.63764 | 10.03930 | 0 | not available | 0.08115 | 0.97915 |
| 714 | 44.63507 | 10.29141 | 2 | 0.69315 | 0.02979 | 0.96051 |
| 715 | 44.63194 | 10.54349 | 4 | 0 | 0.0782 | 0.87455 |
| 716 | 44.62825 | 10.79553 | 3 | 0 | 0.02053 | 0.83642 |
| 717 | 44.62401 | 11.04751 | 5 | 0 | 0.01616 | 0.90515 |
| 718 | 44.61922 | 11.29943 | 0 | not available | 0.01514 | 0.89023 |
| 719 | 44.61387 | 11.55129 | 0 | not available | 0.11054 | 0.96831 |
| 720 | 44.60796 | 11.80307 | 0 | not available | 0.20547 | 0.98072 |
| 721 | 44.60150 | 12.05477 | 0 | not available | 0.54356 | 0.99378 |
| 722 | 44.59449 | 12.30638 | 0 | not available | 0.28418 | 0.98226 |
| 723 | 44.80034 | 6.54573 | 0 | not available | 0.01198 | 0.05066 |
| 724 | 44.80503 | 7.07291 | 0 | not available | 0.13443 | 0.59186 |
| 725 | 44.80916 | 7.60067 | 0 | not available | 0.00536 | 0.95033 |
| 726 | 44.81273 | 7.12895 | 24 | 0 | 0.00817 | 0.96828 |
| 727 | 44.81574 | 7.65765 | 16 | 0 | 0.14436 | 0.95707 |
| 728 | 44.81820 | 8.18672 | 2 | 0 | 0.00892 | 0.96788 |
| 729 | 44.82009 | 8.71607 | 6 | 0.45056 | 0.02365 | 0.95804 |
| 730 | 44.82142 | 8.24564 | 2 | 0 | 0.05138 | 0.95675 |
| 731 | 44.82220 | 8.77534 | 0 | not available | 0.06043 | 0.91258 |
| 732 | 44.82241 | 9.30633 | 0 | not available | 0.01198 | 0.99221 |
| 733 | 44.82206 | 9.83684 | 0 | not available | 0.01619 | 0.99207 |
| 734 | 44.82115 | 9.36652 | 0 | not available | 0.16371 | 0.98761 |
| 735 | 44.81968 | 9.89604 | 0 | not available | 0.02832 | 0.98685 |
| 736 | 44.81766 | 10.04253 | 0 | not available | 0.05924 | 0.96007 |
| 737** | 44.81507 | 10.29543 | 8 | 1.66746 | 0.08898 | 0.86348 |
| 738 | 44.81192 | 10.54829 | 2 | 0.69315 | 0.02096 | 0.92327 |
| 739 | 44.80821 | 10.80110 | 1 | not available | 0.09494 | 0.91375 |
| 740 | 44.80394 | 11.05387 | 1 | not available | 0.0209 | 0.93924 |
| 741 | 44.79912 | 11.30657 | 0 | not available | 0.0364 | 0.95285 |
| 742 | 44.79373 | 11.55921 | 1 | not available | 0.02303 | 0.89681 |
| 743 | 44.78779 | 11.81177 | 0 | not available | 0.02197 | 0.98162 |
| 744 | 44.78129 | 12.06425 | 0 | not available | 0.17799 | 0.97369 |
| 745 | 44.77423 | 12.31664 | 0 | not available | 0.31561 | 0.97259 |
| 746 | 44.76662 | 12.56893 | 0 | not available | 0.0062 | 0.99994 |
| 747 | 44.98024 | 6.47558 | 0 | not available | 0.12564 | 0.55477 |
| 748 | 44.98496 | 7.01064 | 0 | not available | 0.39705 | 0.99377 |
| 749 | 44.98912 | 7.54629 | 0 | not available | 0.1529 | 0.95763 |
| 750 | 44.99271 | 7.08246 | 7 | 0.68291 | 0.06196 | 0.77879 |
| 751 | 44.99574 | 7.61906 | 27 | 0.15841 | 0.06687 | 0.77615 |
| 752 | 44.99821 | 8.15603 | 2 | 0 | 0.0286 | 0.98185 |
| 753 | 45.00012 | 8.69329 | 0 | not available | 0.0501 | 0.98496 |
| 754 | 45.00146 | 8.23076 | 0 | not available | 0.05393 | 0.96368 |
| 755 | 45.00223 | 8.76838 | 0 | not available | 0.30387 | 0.97581 |
| 756 | 45.00245 | 9.30729 | 1 | not available | 0.00035 | 0.92786 |
| 757 | 45.00210 | 9.84572 | 0 | not available | 0.00122 | 0.96591 |
| 758 | 45.00119 | 9.38330 | 0 | not available | 0.04806 | 0.93093 |
| 759 | 44.99971 | 9.92073 | 1 | not available | 0.059 | 0.88905 |
| 760 | 44.99767 | 10.04579 | 0 | not available | 0.04489 | 0.97496 |
| 761 | 44.99506 | 10.29948 | 1 | not available | 0.12262 | 0.95092 |
| 762 | 44.99189 | 10.55313 | 2 | 0 | 0.15248 | 0.92836 |
| 763 | 44.98816 | 10.80673 | 0 | not available | 0.09116 | 0.93824 |
| 764 | 44.98387 | 11.06028 | 0 | not available | 0.03305 | 0.95357 |
| 765 | 44.97901 | 11.31378 | 2 | 0 | 0.08343 | 0.96265 |
| 766 | 44.97360 | 11.56720 | 0 | not available | 0.04696 | 0.95711 |
| 767 | 44.96762 | 11.82055 | 0 | not available | 0.03563 | 0.94779 |
| 768 | 44.96108 | 12.07381 | 0 | not available | 0.07906 | 0.96896 |
| 769 | 44.95397 | 12.32698 | 0 | not available | 0.31731 | 0.95757 |
| 770 | 44.94631 | 12.58006 | 0 | not available | 0.13435 | 0.98605 |
| 771 | 45.16014 | 6.40476 | 0 | not available | 0.06899 | 0.34447 |
| 772 | 45.16489 | 6.94778 | 0 | not available | 0.13737 | 0.67518 |
| 773 | 45.16907 | 7.49140 | 0 | not available | 0.05563 | 0.96713 |
| 774 | 45.17269 | 7.03553 | 0 | not available | 0.22752 | 0.87235 |
| 775 | 45.17574 | 7.58011 | 3 | 0 | 0.06188 | 0.7791 |
| 776 | 45.17822 | 8.12505 | 8 | 0 | 0.10258 | 0.95163 |
| 777 | 45.18014 | 8.67029 | 0 | not available | 0.17184 | 0.97254 |
| 778 | 45.18149 | 8.21575 | 1 | not available | 0.38667 | 0.94907 |
| 779 | 45.18227 | 8.76135 | 0 | not available | 0.26478 | 0.94863 |
| 780 | 45.18248 | 9.30826 | 0 | not available | 0.19065 | 0.92362 |
| 781 | 45.18213 | 9.85468 | 0 | not available | 0.08339 | 0.92238 |
| 782 | 45.18121 | 9.40025 | 0 | not available | 0.05657 | 0.92656 |
| 783 | 45.17973 | 9.94566 | 0 | not available | 0.06204 | 0.94311 |
| 784 | 45.17767 | 10.04908 | 0 | not available | 0.03749 | 0.91132 |
| 785 | 45.17505 | 10.30357 | 0 | not available | 0.0434 | 0.95629 |
| 786 | 45.17186 | 10.55801 | 0 | not available | 0.03854 | 0.95504 |
| 787 | 45.16811 | 10.81241 | 1 | not available | 0.0668 | 0.88994 |
| 788 | 45.16379 | 11.06676 | 0 | not available | 0.01699 | 0.94394 |
| 789 | 45.15890 | 11.32105 | 0 | not available | 0.02066 | 0.92265 |
| 790 | 45.15345 | 11.57527 | 0 | not available | 0.02077 | 0.92157 |
| 791 | 45.14743 | 11.82941 | 0 | not available | 0.0023 | 0.89768 |
| 792 | 45.14085 | 12.08346 | 0 | not available | 0.00853 | 0.95816 |
| 793 | 45.13371 | 12.33743 | 0 | not available | 0.24405 | 0.96026 |
| 794 | 45.34481 | 6.88433 | 0 | not available | 0.00238 | 0.00205 |
| 795 | 45.34902 | 7.43598 | 0 | not available | 0.14693 | 0.89421 |
| 796 | 45.35266 | 7.98816 | 0 | not available | 0.04211 | 0.97256 |
| 797 | 45.35572 | 7.54078 | 0 | not available | 0.07054 | 0.9338 |
| 798 | 45.35822 | 8.09378 | 0 | not available | 0.03947 | 0.9374 |
| 799 | 45.36015 | 8.64708 | 0 | not available | 0.05501 | 0.96887 |
| 800 | 45.36151 | 8.20059 | 0 | not available | 0.03792 | 0.91543 |
| 801 | 45.36230 | 8.75425 | 0 | not available | 0.11628 | 0.90964 |
| 802 | 45.36251 | 9.30924 | 0 | not available | 0.09685 | 0.83828 |
| 803 | 45.36216 | 9.86372 | 0 | not available | 0.00111 | 0.78292 |
| 804 | 45.36123 | 9.41736 | 0 | not available | 0.02881 | 0.90614 |
| 805 | 45.35974 | 9.97083 | 1 | not available | 0.02627 | 0.92685 |
| 806 | 45.35767 | 10.05240 | 0 | not available | 0.00357 | 0.9218 |
| 807 | 45.35503 | 10.30769 | 0 | not available | not available | 0.89338 |
| 808 | 45.35183 | 10.56295 | 0 | not available | 0.00847 | 0.92131 |
| 809 | 45.34805 | 10.81815 | 0 | not available | 0.00295 | 0.87207 |
| 810 | 45.34370 | 11.07330 | 0 | not available | 0.02549 | 0.84038 |
| 811 | 45.33878 | 11.32839 | 0 | not available | 0.06811 | 0.90478 |
| 812 | 45.33330 | 11.58341 | 0 | not available | 0.30795 | 0.957 |
| 813 | 45.32724 | 11.83835 | 0 | not available | 0.14988 | 0.79658 |
| 814 | 45.32062 | 12.09321 | 0 | not available | 0.31177 | 0.92736 |
| 815 | 45.31343 | 12.34798 | 0 | not available | 0.38182 | 0.98309 |
| 816 | 45.52472 | 6.82028 | 0 | not available | 0.08703 | 0.34057 |
| 817** | 45.52895 | 7.38004 | 1 | not available | 0.9436 | 0.99852 |
| 818** | 45.53262 | 7.94034 | 0 | not available | 0.70486 | 1 |
| 819** | 45.53571 | 7.50109 | 29 | 2.13913 | 0.12962 | 0.97103 |
| 820 | 45.53822 | 8.06222 | 2 | 0 | 0.17399 | 0.89096 |
| 821 | 45.54016 | 8.62364 | 0 | not available | 0.07077 | 0.92968 |
| 822 | 45.54153 | 8.18530 | 0 | not available | 0.04032 | 0.92008 |
| 823 | 45.54232 | 8.74709 | 0 | not available | 0.1832 | 0.72149 |
| 824 | 45.54254 | 9.31023 | 0 | not available | 0.0149 | 0.49307 |
| 825 | 45.54218 | 9.87286 | 0 | not available | 0.02701 | 0.38871 |
| 826 | 45.54125 | 9.43462 | 1 | not available | 0.0143 | 0.80749 |
| 827 | 45.53974 | 9.99623 | 0 | not available | 0.00013 | 0.8884 |
| 828 | 45.53767 | 10.05575 | 0 | not available | not available | 0.81671 |
| 829 | 45.53501 | 10.31186 | 0 | not available | 0.01392 | 0.77602 |
| 830 | 45.53178 | 10.56792 | 0 | not available | 0.03396 | 0.88697 |
| 831 | 45.52798 | 10.82394 | 2 | 0 | 0.1025 | 0.84776 |
| 832 | 45.52361 | 11.07990 | 1 | not available | 0.03433 | 0.91673 |
| 833 | 45.51866 | 11.33580 | 0 | not available | 0.01767 | 0.87631 |
| 834 | 45.51314 | 11.59163 | 0 | not available | 0.18383 | 0.86757 |
| 835 | 45.50705 | 11.84738 | 0 | not available | 0.05337 | 0.84965 |
| 836 | 45.50038 | 12.10305 | 0 | not available | 0.01067 | 0.80867 |
| 837 | 45.49315 | 12.35862 | 0 | not available | 0.4231 | 0.84291 |
| 838 | 45.48534 | 12.61409 | 0 | not available | 0.23199 | 0.96115 |
| 839 | 45.47696 | 12.86945 | 0 | not available | 0.00269 | 0.99408 |
| 840 | 45.69979 | 6.18828 | 0 | not available | 0.04109 | 0.06851 |
| 841 | 45.70463 | 6.75562 | 7 | 0.79631 | 0.04659 | 0.83583 |
| 842** | 45.70889 | 7.32357 | 70 | 2.05061 | 0.19855 | 0.95767 |
| 843*** | 45.71257 | 7.89206 | 17 | 1.78902 | 0.47642 | 0.98178 |
| 844** | 45.71568 | 7.46101 | 25 | 1.10269 | 0.03445 | 0.98038 |
| 845 | 45.71821 | 8.03035 | 0 | not available | 0.5211 | 0.99758 |
| 846 | 45.72016 | 8.59999 | 0 | not available | 0.0945 | 0.94396 |
| 847 | 45.72154 | 8.16985 | 0 | not available | 0.07712 | 0.86674 |
| 848 | 45.72234 | 8.73986 | 0 | not available | 0.13774 | 0.63723 |
| 849 | 45.72256 | 9.31122 | 0 | not available | 0.11947 | 0.63639 |
| 850 | 45.72220 | 9.88207 | 0 | not available | 0.14236 | 0.68036 |
| 851 | 45.72126 | 9.45206 | 0 | not available | 0.04694 | 0.74797 |
| 852 | 45.71975 | 9.02187 | 0 | not available | 0.00629 | 0.79025 |
| 853 | 45.71765 | 10.05914 | 0 | not available | 0.01156 | 0.93441 |
| 854 | 45.71498 | 10.31606 | 0 | not available | 0.00178 | 0.9459 |
| 855 | 45.71173 | 10.57295 | 0 | not available | 0.3753 | 0.97763 |
| 856 | 45.70791 | 10.82979 | 0 | not available | 0.3631 | 0.97698 |
| 857 | 45.70351 | 11.08657 | 1 | not available | 0.42231 | 0.98988 |
| 858 | 45.69853 | 11.34329 | 2 | 0 | 0.09165 | 0.87554 |
| 859 | 45.69297 | 11.59993 | 1 | not available | 0.03961 | 0.86137 |
| 860 | 45.68684 | 11.85650 | 0 | not available | 0.05921 | 0.83693 |
| 861 | 45.68014 | 12.11298 | 0 | not available | 0.04431 | 0.86486 |
| 862 | 45.67286 | 12.36937 | 0 | not available | 0.05997 | 0.87114 |
| 863 | 45.66500 | 12.62565 | 0 | not available | 0.00305 | 0.92099 |
| 864 | 45.65657 | 12.88183 | 0 | not available | 0.11972 | 0.96878 |
| 865 | 45.64757 | 13.13788 | 0 | not available | 0.17286 | 0.95695 |
| 866 | 45.63800 | 13.39381 | 0 | not available | 0.17274 | 0.99183 |
| 867 | 45.62785 | 13.64961 | 0 | not available | 0.02505 | 0.89592 |
| 868 | 45.61714 | 13.90527 | 0 | not available | 0.08344 | 0.16768 |
| 869 | 45.87966 | 6.11474 | 0 | not available | 0.03123 | 0.03114 |
| 870 | 45.88453 | 6.69034 | 0 | not available | 0.32276 | 0.42206 |
| 871 | 45.88881 | 7.26656 | 4 | 0.69315 | 0.01892 | 0.49379 |
| 872 | 45.89252 | 7.84333 | 1 | not available | 0.01189 | 0.77993 |
| 873 | 45.89565 | 7.42056 | 0 | not available | 0.22941 | 0.74947 |
| 874 | 45.89820 | 7.99818 | 0 | not available | 0.45565 | 0.98756 |
| 875 | 45.90016 | 8.57610 | 0 | not available | 0.1272 | 0.99373 |
| 876 | 45.90155 | 8.15426 | 0 | not available | 0.05418 | 0.87829 |
| 877 | 45.90235 | 8.73256 | 0 | not available | 0.15833 | 0.74576 |
| 878 | 45.90257 | 9.31223 | 0 | not available | 0.01622 | 0.50813 |
| 879 | 45.90221 | 9.89138 | 0 | not available | 0.21425 | 0.89238 |
| 880 | 45.90126 | 9.46966 | 0 | not available | 0.22892 | 0.9626 |
| 881 | 45.89974 | 9.04776 | 0 | not available | 0.27779 | 0.96927 |
| 882 | 45.89763 | 10.06256 | 0 | not available | 0.30528 | 0.93569 |
| 883 | 45.89495 | 10.32031 | 0 | not available | 0.17676 | 0.97105 |
| 884 | 45.89168 | 10.57802 | 1 | not available | 0.23104 | 0.98781 |
| 885 | 45.88783 | 10.83569 | 1 | not available | 0.16677 | 0.9652 |
| 886 | 45.88340 | 11.09330 | 6 | 0 | 0.1445 | 0.95409 |
| 887 | 45.87839 | 11.35084 | 0 | not available | 0.21012 | 0.97961 |
| 888 | 45.87280 | 11.60831 | 0 | not available | 0.23452 | 0.96592 |
| 889 | 45.86663 | 11.86570 | 0 | not available | 0.49412 | 0.94655 |
| 890 | 45.85988 | 12.12301 | 0 | not available | 0.23399 | 0.89983 |
| 891 | 45.85256 | 12.38021 | 0 | not available | 0.06822 | 0.87354 |
| 892 | 45.84465 | 12.63732 | 0 | not available | 0.03048 | 0.90193 |
| 893 | 45.83617 | 12.89431 | 0 | not available | 0.01805 | 0.90249 |
| 894 | 45.82711 | 13.15119 | 2 | 0.69315 | 0.12646 | 0.93215 |
| 895 | 45.81748 | 13.40794 | 0 | not available | 0.09436 | 0.87719 |
| 896 | 45.80727 | 13.66455 | 0 | not available | 0.20483 | 0.44823 |
| 897 | 45.79649 | 13.92102 | 0 | not available | 0.01403 | 0.01601 |
| 898 | 46.07247 | 7.79413 | 0 | not available | not available | 0.00211 |
| 899 | 46.07818 | 7.96570 | 0 | not available | 0.28384 | 0.29475 |
| 900 | 46.08015 | 8.55199 | 0 | not available | 0.373 | 0.94121 |
| 901 | 46.08155 | 8.13852 | 0 | not available | 0.29654 | 0.94187 |
| 902 | 46.08235 | 8.72519 | 0 | not available | 0.12299 | 0.41161 |
| 903 | 46.08257 | 9.31324 | 0 | not available | 0.01088 | 0.33457 |
| 904 | 46.08221 | 9.90078 | 0 | not available | 0.0234 | 0.95404 |
| 905 | 46.08126 | 9.48742 | 0 | not available | 0.35157 | 0.96645 |
| 906 | 46.07973 | 9.07390 | 0 | not available | 0.47648 | 0.98321 |
| 907 | 46.07761 | 10.06601 | 0 | not available | 0.57313 | 0.99129 |
| 908 | 46.07491 | 10.32460 | 0 | not available | 0.28144 | 0.98442 |
| 909** | 46.07162 | 10.58315 | 0 | not available | 0.63871 | 0.99787 |
| 910 | 46.06774 | 10.84165 | 0 | not available | 0.2742 | 0.97656 |
| 911 | 46.06329 | 11.10009 | 11 | 0 | 0.04326 | 0.89184 |
| 912 | 46.05825 | 11.35847 | 7 | 0.41012 | 0.11308 | 0.95461 |
| 913 | 46.05262 | 11.61677 | 0 | not available | 0.32096 | 0.98068 |
| 914 | 46.04641 | 11.87499 | 0 | not available | 0.51525 | 0.96822 |
| 915 | 46.03962 | 12.13313 | 0 | not available | 0.23891 | 0.9607 |
| 916 | 46.03225 | 12.39117 | 2 | 0 | 0.2603 | 0.92316 |
| 917 | 46.02430 | 12.64910 | 1 | not available | 0.11006 | 0.84716 |
| 918 | 46.01576 | 12.90692 | 1 | not available | 0.06342 | 0.91007 |
| 919 | 46.00665 | 13.16462 | 5 | 0.67301 | 0.01155 | 0.81986 |
| 920 | 45.99696 | 13.42219 | 2 | 0.69315 | 0.01796 | 0.79231 |
| 921 | 45.98668 | 13.67963 | 6 | 1.0114 | not available | 0.11264 |
| 922 | 46.25815 | 7.93291 | 0 | not available | 0.02473 | 0.02449 |
| 923 | 46.26014 | 8.52765 | 0 | not available | 0.36703 | 0.85265 |
| 924 | 46.26154 | 8.12263 | 0 | not available | 0.22898 | 0.37514 |
| 925 | 46.26221 | 9.91026 | 0 | not available | 0.15308 | 0.64244 |
| 926 | 46.26126 | 9.50536 | 0 | not available | 0.25723 | 0.80467 |
| 927 | 46.25971 | 9.10028 | 0 | not available | 0.30524 | 0.91752 |
| 928 | 46.25758 | 10.06949 | 0 | not available | 0.24648 | 0.66054 |
| 929 | 46.25486 | 10.32892 | 0 | not available | 0.2847 | 0.98774 |
| 930 | 46.25155 | 10.58832 | 0 | not available | 0.62728 | 0.99297 |
| 931** | 46.24765 | 10.84766 | 0 | not available | 0.73159 | 0.98596 |
| 932 | 46.24317 | 11.10694 | 4 | 0 | 0.15408 | 0.95722 |
| 933 | 46.23809 | 11.36616 | 2 | 0 | 0.31399 | 0.97932 |
| 934 | 46.23243 | 11.62531 | 0 | not available | 0.59425 | 0.98779 |
| 935 | 46.22619 | 11.88437 | 0 | not available | 0.55286 | 0.98838 |
| 936 | 46.21936 | 12.14335 | 0 | not available | 0.52782 | 0.97193 |
| 937 | 46.21194 | 12.40222 | 1 | not available | 0.16021 | 0.97435 |
| 938 | 46.20393 | 12.66099 | 0 | not available | 0.23604 | 0.96728 |
| 939*** | 46.19535 | 12.91965 | 9 | 1.42706 | 0.21402 | 0.94057 |
| 940 | 46.18618 | 13.17818 | 3 | 1.09861 | 0.03409 | 0.85939 |
| 941 | 46.17642 | 13.43659 | 9 | 0 | 0.05165 | 0.80606 |
| 942 | 46.16609 | 13.69485 | 0 | not available | 0.00009 | 0.14492 |
| 943 | 46.44012 | 8.50308 | 0 | not available | 0.15496 | 0.15421 |
| 944 | 46.44153 | 8.10658 | 0 | not available | 0.17379 | 0.18977 |
| 945 | 46.44220 | 9.91984 | 0 | not available | 0.03381 | 0.44766 |
| 946 | 46.44124 | 9.52347 | 0 | not available | 0.00091 | 0.13604 |
| 947 | 46.43969 | 9.12692 | 0 | not available | 0.04842 | 0.04853 |
| 948 | 46.43754 | 10.07301 | 0 | not available | 0.25728 | 0.45234 |
| 949 | 46.43481 | 10.33329 | 0 | not available | 0.52393 | 0.98596 |
| 950** | 46.43148 | 10.59354 | 0 | not available | 0.96852 | 0.99752 |
| 951 | 46.42755 | 10.85373 | 0 | not available | 0.33445 | 0.99499 |
| 952 | 46.42304 | 11.11387 | 2 | 0.69315 | 0.04926 | 0.97049 |
| 953 | 46.41794 | 11.37394 | 0 | not available | 0.02321 | 0.93409 |
| 954 | 46.41224 | 11.63393 | 0 | not available | 0.22211 | 0.98727 |
| 955 | 46.40596 | 11.89384 | 0 | not available | 0.22531 | 0.98956 |
| 956 | 46.39908 | 12.15366 | 0 | not available | 0.5368 | 0.99207 |
| 957** | 46.39162 | 12.41338 | 0 | not available | 0.81984 | 0.98795 |
| 958 | 46.38356 | 12.67300 | 2 | 0 | 0.48052 | 0.99669 |
| 959 | 46.37492 | 12.93250 | 5 | 0.5004 | 0.07477 | 0.97988 |
| 960** | 46.36569 | 13.19187 | 7 | 0.41012 | 0.19399 | 0.97735 |
| 961 | 46.35588 | 13.45112 | 0 | not available | 0.2799 | 0.60383 |
| 962 | 46.34548 | 13.71022 | 0 | not available | not available | 0.00112 |
| 963 | 46.61750 | 10.07656 | 0 | not available | 0.17297 | 0.23231 |
| 964 | 46.61475 | 10.33770 | 0 | not available | 0.21895 | 0.32464 |
| 965 | 46.61140 | 10.59881 | 0 | not available | 0.59141 | 0.96163 |
| 966 | 46.60745 | 10.85986 | 1 | not available | 0.32529 | 0.99312 |
| 967 | 46.60291 | 11.12086 | 1 | not available | 0.02367 | 0.95282 |
| 968 | 46.59777 | 11.38178 | 0 | not available | 0.0001 | 0.99552 |
| 969 | 46.59204 | 11.64263 | 0 | not available | 0.15694 | 0.98117 |
| 970 | 46.58572 | 11.90340 | 0 | not available | 0.52089 | 0.99311 |
| 971** | 46.57880 | 12.16408 | 0 | not available | 0.67879 | 0.99208 |
| 972** | 46.57129 | 12.42465 | 0 | not available | 0.76468 | 0.98759 |
| 973** | 46.56318 | 12.68512 | 4 | 0 | 0.66635 | 0.96801 |
| 974 | 46.55449 | 12.94547 | 2 | 0 | 0.20043 | 0.77061 |
| 975 | 46.54520 | 13.20570 | 0 | not available | 0.20766 | 0.65312 |
| 976 | 46.53533 | 13.46579 | 1 | not available | 0.18698 | 0.62361 |
| 977 | 46.52486 | 13.72574 | 0 | not available | 0.09022 | 0.21298 |
| 978 | 46.79468 | 10.34216 | 0 | not available | not available | 0.11179 |
| 979 | 46.79131 | 10.60413 | 0 | not available | 0.01754 | 0.84575 |
| 980 | 46.78734 | 10.86605 | 0 | not available | 0.20352 | 0.45412 |
| 981 | 46.78277 | 11.12791 | 0 | not available | 0.50223 | 0.85768 |
| 982 | 46.77760 | 11.38971 | 0 | not available | 0.00041 | 1 |
| 983 | 46.77183 | 11.65142 | 0 | not available | 0.00063 | 0.9788 |
| 984 | 46.76547 | 11.91305 | 0 | not available | 0.16139 | 0.97287 |
| 985 | 46.75851 | 12.17459 | 0 | not available | 0.28923 | 0.96566 |
| 986 | 46.75095 | 12.43603 | 0 | not available | 0.09765 | 0.26896 |
| 987 | 46.74279 | 12.69736 | 0 | not available | 0.00584 | 0.00468 |
| 988 | 46.96262 | 11.13504 | 0 | not available | 0.03263 | 0.28794 |
| 989 | 46.95742 | 11.39771 | 0 | not available | 0.00374 | 0.64967 |
| 990 | 46.95162 | 11.66030 | 0 | not available | 0.0002 | 0.7242 |
| 991 | 46.94522 | 11.92280 | 0 | not available | 0.24811 | 0.91093 |
| 992 | 46.93821 | 12.18521 | 0 | not available | 0.32989 | 0.48307 |
| 993 | 47.12495 | 11.93264 | 0 | not available | not available | 0.03978 |
| 994 | 47.11790 | 12.19593 | 0 | not available | 0.11174 | 0.16794 |
