# Supplementary material for: A methodological approach to identify agro-biodiversity hotspots for priority in situ conservation of plant genetic resources
Source: PLoS One. 2018 Jun 1;13(6):e0197709. doi: 10.1371/journal.pone.0197709 (PMC5983459; doi:10.1371/journal.pone.0197709)
Supplement: S2 Table — (DOCX) [file pone.0197709.s003.docx]

S2 Table. Italian Corine Land Cover Class.

CORINE Land Cover (CLC) is a geographic land cover/land use database encompassing most of the countries of Europe. In 1985 the Corine programme was initiated in the European Union, the Corine databases and several of its programme have been taken over by the EEA. One of these is an inventory of land cover in 44 classes organised hierarchically in three levels, and presented as a cartographic product, at a scale of 1:100 000. The first level (5 classes) corresponds to the main categories of the land cover/land use (artificial areas, agricultural land, forests and semi-natural areas, wetlands, water surfaces). The second level (15 classes) covers physical and physiognomic entities at a higher level of detail (urban zones, forests, lakes, etc), finally the third level is composed of 44 classes. CLC was elaborated based on the visual interpretation of satellite images (SPOT, LANDSAT TM and MSS). Ancillary data (aerial photographs, topographic or vegetation maps, statistics, local knowledge) were used to refine interpretation and the assignment of the territory into the categories of the CORINE Land Cover nomenclature

The smallest surfaces mapped (minimum mapping units) correspond to 25 hectares. Linear features less than 100 m in width are not considered. The scale of the output product was fixed at 1:100.000. Thus, the location precision of the CLC database is 100 m.

This database is operationally available for most areas of Europe. Original inventories, based on and interpreted from satellite imagery as well as ancillary information sources, are stored within national institutions (European Commission 2000).

Source http://sia.eionet.europa.eu/CLC2006/ (accessed at 26 February 2015)

| Level 1 | Level 2 | Level 3 | Grid code |
| --- | --- | --- | --- |
| 1. ARTIFICIAL SURFACES | 1.1 Urban fabric | 1.1.1 Continuous urban fabric | 111 |
|  |  | 1.1.2 Discontinuous urban fabric | 112 |
|  | 1.2 Industrial, commercial and transport units | 1.2.1 Industrial or commercial units | 121 |
|  |  | 1.2.2 Road and rail networks and associated land | 122 |
|  |  | 1.2.3 Port areas | 123 |
|  |  | 1.2.4 Airports | 124 |
|  | 1.3 Mine, dump and construction sites | 1.3.1 Mineral extraction sites | 131 |
|  |  | 1.3.2 Dump sites | 132 |
|  |  | 1.3.3 Construction sites | 133 |
|  | 1.4 Artificial, non-agricultural vegetated areas | 1.4.1 Green urban areas | 141 |
|  |  | 1.4.2 Sport and leisure facilities | 142 |
|  |  |  |  |
| 2. AGRICULTURAL AREAS | 2.1 Arable land | 2.1.1 Non-irrigated arable land | 211 |
|  |  | 2.1.2 Permanently irrigated land | 212 |
|  |  | 2.1.3 Rice fields | 213 |
|  | 2.2 Permanent crops | 2.2.1 Vineyards | 221 |
|  |  | 2.2.2 Fruit trees and berry plantations | 222 |
|  |  | 2.2.3 Olive groves | 223 |
|  | 2.3 Pastures | 2.3.1 Pastures | 231 |
|  | 2.4 Heterogeneous agricultural areas | 2.4.1 Annual crops associated with permanent crops | 241 |
|  |  | 2.4.2 Complex cultivation patterns | 242 |
|  |  | 2.4.3 Land principally occupied by agriculture, with significant areas of natural vegetation | 243 |
|  |  | 2.4.4 Agro-forestry areas | 244 |

| Level 1 | Level 2 | Level 3 | Grid code |
| --- | --- | --- | --- |
| 3. FOREST AND SEMI NATURAL AREAS | 3.1 Forests | 3.1.1 Broad-leaved forest | 311 |
|  |  | 3.1.2 Coniferous forest | 312 |
|  |  | 3.1.3 Mixed forest | 313 |
|  | 3.2 Scrub and/or herbaceous vegetation associations | 3.2.1 Natural grasslands | 321 |
|  |  | 3.2.2 Moors and heathland | 322 |
|  |  | 3.2.3 Sclerophyllous vegetation | 323 |
|  |  | 3.2.4 Transitional woodland-shrub | 324 |
|  | 3.3 Open spaces with little or no vegetation | 3.3.1 Beaches, dunes, sands | 331 |
|  |  | 3.3.2 Bare rocks | 332 |
|  |  | 3.3.3 Sparsely vegetated areas | 333 |
|  |  | 3.3.4 Burnt areas | 334 |
|  |  | 3.3.5 Glaciers and perpetual snow | 335 |
|  |  |  |  |
| 4. WETLANDS | 4.1 Inland wetlands | 4.1.1 Inland marshes | 411 |
|  |  | 4.1.2 Peat bogs | 412 |
|  | 4.2 Maritime wetlands | 4.2.1 Salt marshes | 421 |
|  |  | 4.2.2 Salines | 422 |
|  |  | 4.2.3 Intertidal flats | 423 |
|  |  |  |  |
| 5. WATER BODIES | 5.1 Inland waters | 5.1.1 Water courses | 511 |
|  |  | 5.1.2 Water bodies | 512 |
|  | 5.2 Marine waters | 5.2.1 Coastal lagoons | 521 |
|  |  | 5.2.2 Estuaries | 522 |
|  |  | 5.2.3 Sea and ocean | 523 |
